# Immunogenicity of BNT162b2, BBIBP-CorV, Gam-COVID-Vac and ChAdOx1 nCoV-19 Vaccines Six Months after the Second Dose: A Longitudinal Prospective Study

**DOI:** 10.3390/vaccines11010056

**Published:** 2022-12-26

**Authors:** Vladimir Petrović, Vladimir Vuković, Aleksandra Patić, Miloš Marković, Mioljub Ristić

**Affiliations:** 1 Department of Epidemiology, Faculty of Medicine, University of Novi Sad, 21000 Novi Sad, Serbia; 2 Institute of Public Health of Vojvodina, 21000 Novi Sad, Serbia; 3 Department of Microbiology with Parasitology and Immunology, Faculty of Medicine, University of Novi Sad, 21000 Novi Sad, Serbia; 4 Department of Immunology, Faculty of Medicine, Institute of Microbiology and Immunology, University of Belgrade, 11000 Belgrade, Serbia

**Keywords:** COVID-19, immunogenicity, vaccine, BNT162b2, BBIBP-CorV, Gam-COVID-Vac, ChAdOx1 nCoV-19

## Abstract

Many available SARS-CoV-2 vaccines demonstrated good humoral response, but studies directly comparing their immunogenicity in the general population are lacking. We evaluated the medium–term kinetics of anti-S SARS-CoV-2 antibodies (Abs) at one and six months after the second dose of BNT162b2, BBIBP-CorV, and Gam-COVID-Vac. Immunogenicity at six months was directly compared between BNT162b2, BBIBP-CorV, Gam-COVID-Vac, and ChAdOx1 nCoV-19. Participants ≥ 20 years old from Novi Sad, Serbia, without prior SARS-CoV-2 infection, were included. Anti S1/S2 IgG antibodies were measured using quantitative LIAISON SARS-CoV-2 assay. A total of 368 participants were included: 231 (62.77%) had sera collected at two time points. Two doses of BNT162b2 were received by 37.50% of participants, followed by BBIBP-CorV (22.01%), Gam-COVID-Vac (21.47%), and ChAdOx1 nCoV-19 (19.02%). Mean Ab levels at the 28th day and 6 months were 216.55 (SD = 105.73) AU/mL and 75.68 (SD = 57.30) for BNT162b2, 194.38 (SD = 140.24) and 90.53 (SD = 111.30) for Gam-COVID-Vac, and 72.74 (SD = 80.04) and 24.43 (SD = 38.43) for BBIBP-CorV group (*p* < 0.01, between two time points across all three groups), with a significant difference between women and men (*p* < 0.01, for both sexes). At the sixth month post-vaccination, the highest mean Ab level was detected in Gam-COVID-Vac group (91.28 AU/mL, SD = 95.96), followed by BNT162b2 (85.25 AU/mL, SD = 60.02), ChAdOx1 nCoV-19 (64.22 AU/mL, SD = 65.30), and BBIBP-CorV (25.26 AU/mL, SD = 36.92) (*p* < 0.01). Anti-spike IgG persistence was demonstrated six months post-vaccination with a significant decline in Ab levels. These results suggest a lower protection against SARS-CoV-2 over time. Our findings support the introduction of additional (booster) doses.

## 1. Introduction

The coronavirus disease 2019 (COVID-19) continues to be a significant global health problem [1,2]. Large-scale vaccination is a crucial factor in the effort to control the pandemic and a necessary condition for return to the pre-pandemic normality [3]. Various vaccine production platforms have been used to develop SARS-CoV-2 vaccines, including mRNA, vector and inactivated vaccines [4,5]. To be effective against COVID-19, a vaccine should induce both humoral and cell-mediated immune response and protect vaccinated individuals from clinically manifested disease and severe forms of COVID-19, in particular [6,7,8]. Ideally, they should also prevent SARS-CoV-2 infection and transmission of the virus to another susceptible person [6,8,9].

Although the correlates of protection against COVID-19 have not yet been defined, it is widely accepted that antibodies (Abs) that can bind to spike (S) protein and neutralize viral entry into cells play a major role in the protective immunity against SARS-CoV-2 infection [6,10]. Thus, persistence of high levels of these Abs could confer long-lasting protection, both after infection and vaccination. Regarding the post-vaccination Ab dynamics, some studies have shown that Ab levels generally reached a peak one month after the second dose [11], with a progressive decline at 12 weeks and 6 months, indicating waning of the immune response over time. Indeed, at 6 months after the second dose, median serum anti-S Ab levels were similar to the levels observed in individuals vaccinated with one dose and COVID-19 convalescents [12]. Moreover, some studies have detected a more rapid decline of Ab levels in individuals aged 65 years and older compared to a younger population [13,14]. Therefore, many public health agencies recommended booster vaccinations of individuals using both homologous and heterologous regimens [15,16].

Even though the available SARS-CoV-2 vaccines have demonstrated the ability to produce a good humoral immune response [17], there is still a paucity of real-world studies comparing the immunogenicity of different SARS-CoV-2 vaccines, especially among the general population. Measuring levels of anti-S Abs in individuals vaccinated with different vaccines at the same time points after vaccination, and using the same immunological assay which quantifies S-protein-binding Abs, could enable comparison of the immunogenicity of different vaccines. Most of the available studies performed thus far investigated Ab levels in specific populations, such as health care workers (HCWs) or residents of nursing homes [18,19,20], which might not reflect the real situation in the general population.

Several different SARS-CoV-2 vaccines against COVID-19 are currently available in Serbia, namely Pfizer-BioNTech BNT162b2 (Comirnaty^®^), Sinopharm BBIBP-CorV (Vero Cell^®^), Gamaleya Research Institute Gam-COVID-Vac (Sputnik V^®^), Oxford-AstraZeneca ChAdOx1 nCoV-19 AZD1222 (Vaxzevria^®^), and Moderna mRNA-1273 (Spikevax^®^) [21]. All of the investigated SARS-CoV-2 vaccines have showed high early effectiveness in our population [22], but the vaccine-induced immunity over several months in our population remains largely unknown, particularly because immunogenicity may vary according to the vaccine type, and demographic characteristics of participants such as sex and age. In our previous study, we examined different cohorts of participants recruited from the general population with a wide age range across both sexes and demonstrated a robust immune response 28 days after the second dose of BNT162b2, BBIBP-CorV or Gam-COVID-Vac in the majority of participants [23]. Here, we did a follow-up immunogenicity study using the same age- and sex- matched group of participants, with the aim to evaluate the medium–term kinetics of anti-S SARS-CoV-2 Abs between two time points, at one month and 6 months after the second dose of vaccine, in individuals vaccinated with BNT162b2, BBIBP-CorV and Gam-COVID-Vac vaccines. Additionally, we included ChAdOx1 nCoV-19 vaccine with a different dosage regimen (2 doses 12 weeks apart) and compared its immunogenicity at 6 months after the second dose of vaccine with the other SARS-CoV-2 vaccines, namely BNT162b2, BBIBP-CorV and Gam-COVID-Vac.

## 2. Materials and Methods

### 2.1. Study Cohort

Participants were selected from the database for COVID-19 immunization monitoring, based on the vaccine type that they received, time period that elapsed from receiving the second dose of vaccine, and sex and age category of the participants. 

For the longitudinal assessment of anti-S SARS-CoV-2 Ab levels, all eligible participants were interviewed by phone and scheduled for blood sample collection at two time points: the 28th day and at 6 months (180 ± 15 days) following the second dose of vaccine.

In order to directly compare immunogenicity between different vaccine platforms 6 months after the second dose, we recruited participants matched by sex and age (10-year age-category groups and those ≥70 years), specifically, 70 participants vaccinated with BNT162b2, 70 with BBIBP-CorV, 70 with Gam-COVID-Vac and 70 with ChAdOx1 nCoV-19. All vaccinated individuals involved in this study received both doses of the same type of vaccine (homologous two-dose regimen) and followed the recommended time schedule between the doses (21 days apart for BNT162b2, BBIBP-CorV and Gam-COVID-Vac vaccines, and 12 weeks apart for the ChAdOx1 nCoV-19 vaccine).

#### Inclusion and Exclusion Criteria 

We included only healthy individuals age ≥ 20 years from the municipality of Novi Sad, Serbia, without evidence of prior laboratory-confirmed SARS-CoV-2 infection, who received two doses of SARS-CoV-2 vaccine in the period January–June 2021 (during the predominance of the Alpha variant of the SARS-CoV-2 in our territory [24]).

Exclusion criteria for all participants were age < 20 years, previous laboratory confirmation of COVID-19 either by real-time RT-PCR (reverse transcription polymerase chain reaction) or by RDT-Ag (rapid diagnostic test for detection of SARS-CoV-2 antigen), or presence of any signs or symptoms related to the SARS-CoV-2 infection 10 days before vaccination, in the period between the two doses, and during 28 days or 6 months after the second dose of the received vaccine. Vaccine mRNA-1273 (Spikevax, Moderna) was not available in Serbia before November 2021 and therefore it was not included in the current study.

### 2.2. Sample Collection and Measurement of IgG Antibody Levels against SARS-CoV-2 Spike Protein

Blood samples were collected and analyzed at the Centre for Virology of the Institute of Public Health of Vojvodina (IPHV), Novi Sad. Serum was tested using the assay that measures the total amount of Abs against the S protein as the primary target of neutralizing antibodies. Quantitative LIAISON SARS-CoV-2 S1/S2 IgG assay (DiaSorin, Saluggia, Italy) which can detect and quantify Abs with high sensitivity and specificity using the indirect chemiluminescence immune assay (CLIA) method performed on the LIAISON^®^ XL Analyzer, was used to measure IgG Ab levels against S1 and S2 subunits of spike protein in each sample [25]. Concentration of SARS-CoV-2 S1/S2 IgG Ab is automatically calculated by the analyzer in arbitrary units (AU/mL) based on a standardized master curve, and the results are graded. 

As we previously described [23], samples with IgG Ab levels below 12.0 AU/mL were considered seronegative, those with levels ≥ 12.0 AU/mL and <15.0 AU/mL were considered equivocal, and samples with the Ab values ≥ 15.0 AU/mL were considered seropositive, as recommended by the manufacturer [25]. Quantification range for the test was 3.8–400.0 AU/mL. As described by the manufacturer, the obtained Ab test results were 94.4% in positive agreement with the results of the Plaque Reduction Neutralization Test (PRNT), which is the gold standard for evaluating the neutralization capacity of Ab against SARS-CoV-2. Neutralizing Abs target both S1 and S2 proteins, so the likelihood of concordance with a neutralization assay was significantly higher when using both of these antigens in testing [25].

### 2.3. Statistical Analyses

Descriptive statistics using absolute frequencies and percentage (%) for categorical variables and mean with the standard deviation (SD) and median with interquartile range (IQR) for continuous variables were used to present data across vaccine groups, stratified by sex and 10-year age categories and those ≥ 70 years were classified in the same group. For statistical analyses and graphical presentation of the data, Ab values below the lower limit of quantitation (3.80 AU/mL) were set to 3.79 whereas those above the upper limit (>400 AU/mL) were set to 401. Percentage change (%-change) in the Ab levels between the two time points for the longitudinal assessment was calculated as the difference in Ab levels measured at 6 months and those measured at the 28th day, divided by the Ab levels at the 28th day, and multiplied by 100.

Pearson’s chi-squared test or Fisher’s exact test for categorical and t-test or analysis of variance (ANOVA) for continuous variables, were used where appropriate. Without assuming the Gaussian distribution, Wilcoxon rank-sum (Fisher’s exact test, where appropriate) or Kruskal–Wallis H test with the Dunn’s pairwise post hoc multiple comparison using Bonferroni adjustment was used to explore differences in the Ab levels between different SARS-CoV-2 vaccine types, sex and age categories. In order to explore potential correlations between Ab levels at different time points and several investigated variables, Spearman’s rank correlation coefficient was used.

Finally, in order to explore the impact of Ab levels measured above the limit of quantitation of the used assay (400.0 AU/mL) on the overall results of this study, we conducted a sensitivity analysis by excluding 28 (12.1%) participants that, at the 28th day after the second dose of vaccine, had the measured Ab levels above the test’s upper limit of quantification, and repeated the statistical analyses. For the second part of the sensitivity analysis that relates to the six months’ post-vaccination time point, only one participant with the measured Ab value above 400 AU/mL at 6 months following vaccination with the Gam-COVID-Vac was excluded. However, in order to avoid the differences between the groups we also excluded three age- and sex- matched participants (one participant from each of the BNT162b2, BBIBP-CorV and ChAdOx1 nCoV-19 vaccine groups).

All statistical analyses and graphical presentation were performed using statistical software package Stata v.16 (StataCorp LLC. 2019). Results at the *p*-value < 0.05 were considered statistically significant.

### 2.4. Ethical Considerations

The Ethics Committee of the Institute of Public Health of Vojvodina, Novi Sad approved the study protocol under the number 01-860/1/2021. In compliance with the national regulations, and considering the necessity to minimize contact time between the medical personnel and the participants during the pandemic, an oral informed consent was obtained from all participants after a detailed explanation of the aims and procedures of the study was given during a telephone interview that preceded sample collection in the recruiting center. All data were anonymized before being accessed and analyzed by the authors.

## 3. Results

**Figure 1:**
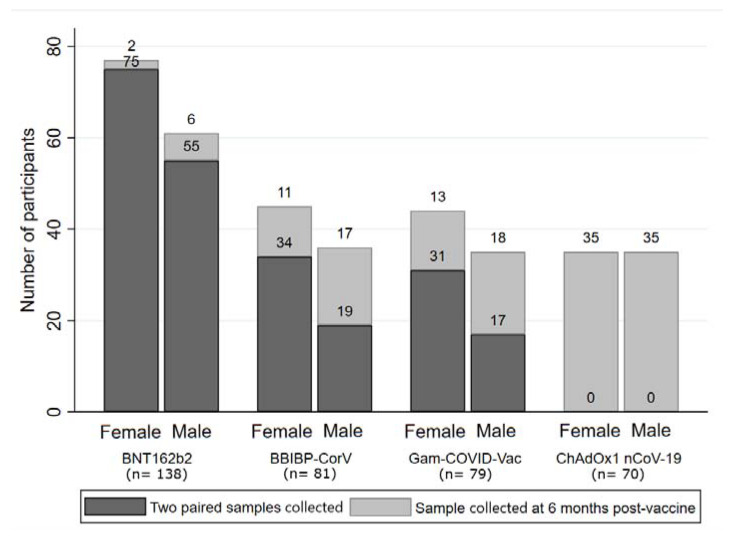
Distribution of participants, stratified by vaccine type, sex and number (single or paired) of samples.

A total of 368 participants vaccinated with two doses of SARS-CoV-2 vaccines were included, of which 231 (62.77%) participants had their sera collected at two time points (paired samples): at the 28th day and after 6 months from the administration of the second dose of vaccine. The majority of participants (*n* = 138, 37.50%) received two doses of BNT162b2, followed by BBIBP-CorV (*n* = 81, 22.01%), Gam-COVID-Vac (*n* = 79, 21.47%), and ChAdOx1 nCoV-19 (*n* = 70, 19.02%) vaccine group (Figure 1).

### 3.1. Longitudinal Course of the SARS-CoV-2 IgG Levels after Vaccination

Out of 231 cohort participants with available two-time point measurements, a majority (*n* = 130, 56.28%) received two doses of BNT162b2, followed by BBIBP-CorV (*n* = 53, 22.94%) and Gam-COVID-Vac (*n* = 48, 20.78%) vaccine. The mean age of participants was 52.48 (SD = 12.99) years, i.e., 53.75 (SD = 13.91) years in the BNT162b2 group, 49.89 (SD = 12.09) years in the BBIBP-CorV group, and 51.9 (SD = 10.96) years in those vaccinated with the Gam-COVID-Vac vaccine (*p* = 0.25). Most of the participants were women (n=75, 57.7%), and regarding three included vaccinated groups (BNT162b2, BBIBP-CorV and Gam-COVID-Vac), they participated with 57.69%, 64.15% and 64.58%, respectively (*p* = 0.59).

Comparison of Ab levels at two time points revealed a significant decline for all vaccine groups, even when stratified by sex and certain age of participants (Table 1).

**Figure 2:**
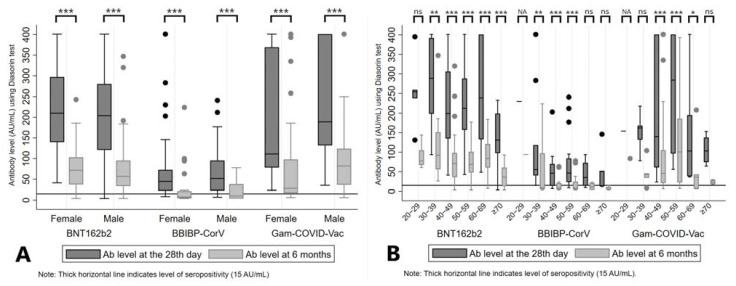
Antibody levels at the 28th day and 6 months after the second dose of vaccine, stratified by sex (**A**) and age groups (**B**) of participants. Legend: *** *p* < 0.001, ** *p* < 0.01, * *p* < 0.05, ns = not significant, NA = not applicable.

Mean Ab levels at the 28th day and at 6 months from administration of the second dose were 216.55 (SD = 105.73) AU/mL and 75.67 (SD = 57.30) AU/mL for BNT162b2, 194.38 (SD = 140.24) AU/mL and 90.53 (SD = 111.30) AU/mL for Gam-COVID-Vac; and 72.74 (SD = 80.04) AU/mL and 24.43 (SD = 38.43) AU/mL for the BBIBP-CorV vaccine group (*p* < 0.01, between two time points across all three groups). Also, there was a significant decrease in Ab levels between two time points regarding sex of the participants, across all three vaccine groups (*p* < 0.01, for both sexes) (Table 1, Figure 2A).

We observed seropositivity in 99.28% and 88.41% of participants at the 28th day and 6 months after the second dose of BNT162b2, 87.65% and 37.04% after BBIBP-CorV, and 100% and 86.08% Gam-COVID-Vac, respectively. These values remained high for BNT162b2 and Gam-COVID-Vac even when stratified by sex (Figure S1, panel A).

After analyzing Ab levels by age groups, a significant difference of measured Ab levels at two time points was reported for BNT162b2 (all age categories ≥ 30 years, *p* < 0.01), BBIBP-CorV (age categories 30 to 59 years, *p* < 0.01) and Gam-COVID-Vac (age categories 40 to 69 years, *p* < 0.05) (Table 1, Figure 2B). A similar pattern was noticed when we analyzed the seropositivity rates, with middle age groups (age categories 30 to 59 years) of those who received BBIBP-CorV and most of the older age categories in BNT162b2 and Gam-COVID-Vac vaccine groups presented with lower proportions of seropositive subjects after 6 months, as demonstrated in Figure S1 (panel B). 

**Figure 3:**
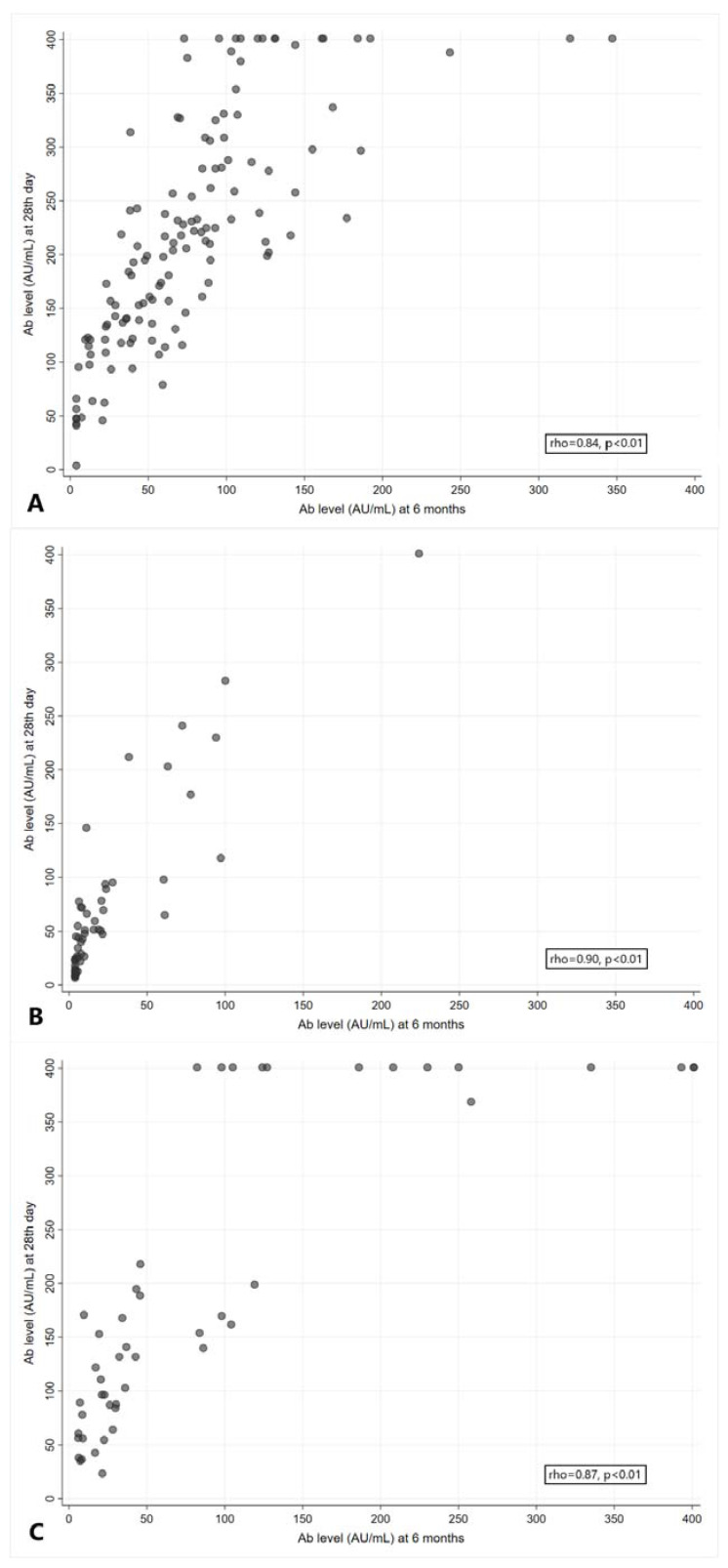
Correlation between Ab levels at the 28th day and 6 months after the second dose of BNT162b2 (**A**), BBIBP-CorV (**B**) and Gam-COVID-Vac (**C**). rho = Spearman’s correlation coefficient.

There was a strong positive correlation between Ab levels at the 28th day and Ab values measured at 6 months after the second dose of BNT162b2 (rho = 0.84, *p* < 0.01), BBIBP-CorV (rho = 0.90, *p* < 0.01) and Gam-COVID-Vac (rho = 0.87, *p* < 0.01), as shown in Figure 3 (panels A, B and C).

We further calculated the percentage change (%-change) of the Ab levels between two measured time points and the lowest mean %-change was for Gam-COVID-Vac (−61.93%, SD = 25.65), followed by BNT162b2 (−66.64%, SD = 17.18) and BBIBP-CorV (−69.47%, SD = 17.22) vaccine (*p* = 0.40). There was no significant (*p* > 0.05) association between calculated %-change regarding sex or age categories of participants in all three vaccine groups (Table 2).

Comparison of the %-change in women and men between three vaccine groups demonstrated equal distribution (*p* = 0.58 and *p* = 0.57, respectively) (Figure S2, panel A). Also, there was no significant difference in %-change of Ab levels (*p* > 0.05) regarding age groups of the participants (Figure S2, panel B). 

Correlation between %-change and Ab levels at first measurement showed statistically significant weak positive correlation in those vaccinated with BNT162b2 (rho = 0.25, *p* < 0.01) and modest positive correlation in the group vaccinated with Gam-COVID-Vac (rho = 0.40, *p* < 0.01), as presented in Figure S3. Similar results were obtained for the correlation at the second measurement after 6 months for BNT162b2 (rho = 0.68, *p* < 0.01) and Gam-COVID-Vac (rho = 0.78, *p* < 0.01), and also for the BBIBP-CorV (rho = 0.28, *p* = 0.04) (Figure S4, panels A, B, and C). On the other hand, correlation with the age groups of participants did not show significant association for either of the investigated vaccine groups, BNT162b2 (rho = −0.05, *p* = 0.56), BBIBP-CorV (rho = −0.16, *p* = 0.25), and Gam-COVID-Vac (rho = −0.24, *p* = 0.10) (Figure S5, panels A, B, and C).

### 3.2. SARS-CoV-2 IgG Levels at Six Months after the Second Dose of Vaccine

In order to be able to directly compare immunogenicity of four SARS-CoV-2 vaccines, 280 matched samples were collected and analyzed, 70 for each of the investigated vaccines, namely BNT162b2, BBIBP-CorV, Gam-COVID-Vac and ChAdOx1 nCoV-19. There were 35 women and 35 men matched by age category across four vaccine groups. Mean age was 49.9 (SD = 15.02) years in the BNT162b2 group, 49.97 (SD = 14.83) years in the BBIBP-CorV, 48.53 (SD = 13.23) years in Gam-COVID-Vac and 48.27 (SD = 14.46) years in those that received ChAdOx1 nCoV-19 vaccine (*p* = 0.84).

**Table 2:** Percentage change in Ab levels between the 28th day and at 6 months, stratified by sex and age groups of participants.

	BNT162b2 Vaccine					BBIBP-CorV Vaccine					Gam-COVID-Vac Vaccine				
	Mean (SD), %-Change	Median, %-Change	IQR (25–75)		*p*-Value ^1^	Mean (SD), %-Change	Median, %-Change	IQR (25–75)		*p*-Value ^1^	Mean (SD), %-Change	Median, %-Change	IQR (25–75)		*p*-Value ^1^
**Total**	−66.64 (17.18)	−68.43	−78.53	−59.34	NA	−69.47 (17.22)	−71.07	−81.12	−60.40	NA	−61.93 (25.65)	−69.57	−79.61	−44.15	NA
**Sex**															
Female	−66.60 (15.66)	−68.72	−79.05	−58.80	0.68	−70.29 (15.36)	−69.61	−80.51	−60.40	0.91	−62.69 (26.73)	−70.07	−81.71	−53.62	0.62
Male	−66.70 (19.21)	−68.35	−78.45	−59.34		−68.00 (20.50)	−73.66	−81.98	−57.42		−60.56 (24.29)	−69.08	−77.90	−42.64	
**Age category**															
20–29	−63.13 (9.89)	−63.54	−69.41	−59.46	0.74	−59.13	−59.13			0.69	−45.65	−45.65			0.47
30–39	−59.87 (20.67)	−67.74	−73.82	−48.36		−63.90 (24.87)	−75.07	−81.12	−44.14		−70.31 (20.74)	−79.04	−79.70	−67.73	
40–49	−68.73 (16.67)	−70.07	−79.27	−64.32		−66.72 (19.14)	−69.28	−78.46	−59.80		−52.39 (30.19)	−61.03	−75.93	−38.50	
50–59	−66.97 (14.90)	−66.77	−78.53	−57.48		−74.37 (12.67)	−76.86	−83.85	−62.95		−62.42 (26.44)	−69.57	−79.53	−40.20	
60–69	−65.95 (14.41)	−72.12	−74.51	−50.15		−69.71 (9.10)	−69.53	−77.37	−62.05		−73.79 (13.65)	−77.90	−83.78	−65.15	
≥70	−67.18 (22.48)	−68.43	−85.11	−58.80		−75.60 (11.06)	−71.07	−80.43	−69.57		−73.68 (15.50)	−75.44	−86.76	−60.60	

Notes: ^1^ Wilcoxon rank-sum or Kruskal–Wallis test; *p*-value refers to difference between the groups of participants vaccinated with the same vaccine. SD = standard deviation, IQR = interquartile range, NA = not applicable.

The highest mean Ab level at 6 months after the second dose was measured in the group vaccinated with Gam-COVID-Vac (91.28 AU/mL, SD = 95.96), followed by BNT162b2 (85.25 AU/mL, SD = 60.02), ChAdOx1 nCoV-19 (64.22 AU/mL, SD = 65.30) and BBIBP-CorV group (25.26 AU/mL, SD = 36.92) (*p* < 0.01) (Table 3). 

Using Dunn’s post hoc test with Bonferroni adjustment, a statistically significant difference was noticed between those vaccinated with BNT162b2 and BBIBP-CorV (*p* < 0.01), BNT162b2 and ChAdOx1 nCoV-19 (*p* = 0.04), and those vaccinated with BBIBP-CorV and Gam-COVID-Vac (*p* < 0.01). Also, Ab levels 6 months after vaccination with ChAdOx1 nCoV-19 were significantly higher compared to BBIBP-CorV vaccine (*p* < 0.01). 

**Figure 4:**
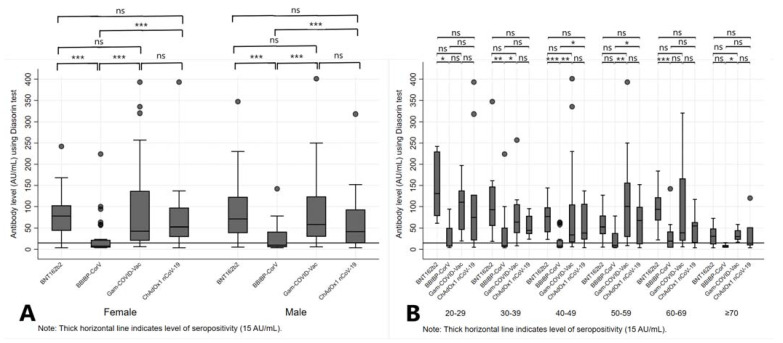
Antibody levels at 6 months after the administration of BNT162b2, BBIBP-CorV, Gam-COVID-Vac and ChAdOx1 nCoV-19 vaccines, stratified by sex (**A**) and age groups (**B**) of participants. Legend: *** *p* < 0.001, ** *p* < 0.01, * *p* < 0.05, ns = not significant.

There was no statistically significant difference in Ab levels regarding sex of the participants within the same vaccine group. However, when we compared Ab levels by sex between four different vaccine groups, we noticed a statistically significant difference (*p* < 0.01 for both sexes). In particular, Ab levels among women and men were significantly higher if they were vaccinated with BNT162b2, Gam-COVID-Vac and ChAdOx1 nCoV-19 vaccines than with BBIBP-CorV (*p* < 0.05) (Figure 4A). The Ab levels varied widely between the investigated age categories across vaccine groups, with significant difference reported only for BNT162b2 group where younger categories were presented with the higher mean Ab levels in respect to the older ones (*p* < 0.01).

Additionally, a statistically significant difference in mean Ab levels was observed among four investigated vaccine groups within the same age category, from categories 30–39 to ≥70 years (*p* < 0.05, across the categories) (Table 3). Results from the post hoc analysis comparing particular vaccine groups within the age category are presented in the Figure 4B. Further, we analyzed seropositivity rates in different vaccine groups 6 months after the second dose and observed that most participants (94.29%) vaccinated with two doses of BNT162b2 remained seropositive (>15.0 AU/mL), compared with 85.71% of participants vaccinated with Gam-COVID-Vac, 78.57% of those vaccinated with the ChAdOx1 nCoV-19 and only 40% of those vaccinated with BBIBP-CorV. Regarding sex of the participants, there was no statistically significant difference (*p* > 0.05) in serological profile at 6 months from the administration of second dose regardless of the vaccine type. Of note, in the group of participants vaccinated with BNT162b2, the mean age was significantly lower in the seropositive compared to the seronegative group (48.88, SD = 14.68 years vs. 66.75, SD = 14.68 years, respectively; *p* = 0.02). We also observed an upkeep of the seropositivity in all participants younger than 50 years at 6 months after the second dose of BNT162b2 vaccine (*p* < 0.01) (Table 4).

Finally, a modest negative correlation was noticed between the age of the participants and Ab levels at 6 months from the administration of the second dose of just BNT162b2 (rho = −0.32, *p* < 0.01), as presented in the Figure S6 (panels A, B, C and D).

### 3.3. Sensitivity Analyses

In order to test the impact of Ab levels measured above the limits of quantitation of the used assay (400.0 AU/mL) on the overall results of this study, we performed sensitivity statistical analysis by limiting our sample to participants whose Ab levels were below 400.0 AU/mL. After excluding 28 (12.1%) participants (14 vaccinated with BNT162b2, 13 with Gam-COVID-Vac, and one with the BBIBP-CorV vaccine) that, at the 28th day after the second dose of vaccine, had the measured Ab levels above the test’s upper limit of quantification (>400 AU/mL), we repeated the statistical analyses on the total sample of 203 participants with the available two time-point paired samples, i.e., 116 (57.14%) vaccinated with BNT162b2, 52 (25.62%) with BBIBP-CorV, and 35 (17.24%) with Gam-COVID-Vac. For the second part of the analyses, after excluding 1 participant with the measured value above 400AU/mL at 6 months following vaccination with Gam-COVID-Vac, we also excluded 3 matching participants by age and sex, from the BNT162b2, BBIBP-CorV and ChAdOx1 nCoV-19 vaccine group. As a result, we were left with 276 participants in total (69 participants per each group). 

Results from our primary analyses were confirmed in the sensitivity analyses, which were not substantially changed, further highlighting the importance of our initial findings (all the results of sensitivity analyses are presented in the Supplementary File S1). We did notice a small reduction in the level of statistical significance across the results probably due to smaller sample size in respect to the initial analyses. The only noticeable change was a somewhat weaker correlation between the %-change and Ab levels at the first measurement with the loss of statistical significance in those vaccinated with Gam-COVID-Vac (rho = 0.17, *p* < 0.34). On the other hand, results remained robust when considering correlation at the second measurement, i.e., 6 months post-vaccination, for BNT162b2 (rho = 0.71, *p* < 0.01), and Gam-COVID-Vac (rho = 0.74, *p* < 0.01), with the loss of statistical significance in the BBIBP-CorV group (rho = 0.24, *p* = 0.09).

## 4. Discussion

With the aim to better understand the longevity of SARS-CoV-2 specific IgG antibodies post-vaccination, we conducted a longitudinal follow-up study among participants vaccinated with four different SARS-CoV-2 vaccines during the Alpha variant of the SARS-CoV-2 predominating in observed population. To the best of our knowledge, this is the first study in our region to provide real-world evidence on the Ab levels at 6 months following the second dose of administrated vaccines, and the percentage change in the Ab levels between the 28th day and 6 months, in the general adult population. In addition, it is one of the rare comparative immunogenicity studies that included both BBIBP-CorV and Gam-COVID-Vac vaccines.

It is already known that levels of neutralizing Abs are helpful parameters in assessing vaccine efficacy, individual and herd immunity against the virus, as well as the durability of humoral immune response, but their protective levels have not yet been defined [6,26]. Therefore, intensive research is focused on neutralizing Ab responses and their utility in gauging COVID-19 vaccine effectiveness (VE) in a real-world situation. In the absence of neutralizing Ab measurements, which so far are the only way to approximate the correlate of protection, Ab tests are accepted as useful indicators of immune protection since Ab levels are much easier to measure than cellular responses, can be performed rapidly and in a high throughput. A number of binding immunoassays and virus neutralization tests have been implemented and cross-validated as a diagnostic tool for determining the level of immune protection [27,28,29]. Several studies reported a significant correlation between the detected levels of anti-S Abs and the serum neutralizing activity tested by neutralization tests. Such correlation suggests a high potential of these tests for quantitative prediction of neutralizing Ab titers and estimation of the level and duration of protection [10,30,31].

Although, seropositivity is not a measure to define the complete level of immune protection, it seems that the presence of circulating Abs against SARS-CoV-2 virus has a significant role in lowering the risk of severe COVID-19, despite the fact that the protective concentration of Ab (correlate of protection) remains unspecified [32]. Therefore, it is plausible to assume that a decrease in Ab titers/levels over time would indicate a sub-optimal protection against SARS-CoV-2 infection [33]. Thus, the assessment of post-vaccination titers/levels can help to better understand vaccine’s long-term efficacy and improve current vaccination strategies [34]. Indeed, serious concerns have been raised about the longevity of post-vaccination immunity, especially with the occurrence of new COVID-19 cases in countries where a high vaccination rate was reached [35,36]. In the longitudinal part of our study, we noticed a significant decline in Ab levels between two time points, at the 28th day and at 6 months, following the application of the second dose of BNT162b2, BBIBP-CorV, and Gam-COVID-Vac. We found that two doses of all three investigated vaccines induced a robust Ab response at the 28th day, with BNT162b2 reaching the highest levels, followed by Gam-COVID-Vac and BBIBP-CorV. The seropositivity to SARS-CoV-2 virus remained at the high level (above 85%) in subjects vaccinated with BNT162b2 and Gam-COVID-Vac at both measurements, but not in those who received BBIBP-CorV, since it dropped below 40% six months after the administration of the second dose of that vaccine. Accordingly, a decrease in Ab levels at 6 months was the most prominent in individuals who received BBIBP-CorV vaccine. Likewise, a significant decline in levels of antibodies specific for the SARS-CoV-2 spike protein receptor binding domain (RBD) three months after vaccination with BBIBP-CorV vaccine has already been reported, with an additional 30% seronegative subjects compared to the 6 weeks’ post-vaccination time-point [37]. Similar to our results, the observed decline after BNT162b2 vaccine during these 3 months was much less prominent in this study. It is noteworthy to mention that the individuals who received Gam-COVID-Vac had slightly higher mean values of Ab levels compared to those who received BNT162b2 vaccine. Similar findings of a significant decrease in Ab levels at 6 months after vaccination against COVID-19 were also reported by other authors [20,38]. In particular, a recent longitudinal prospective study from Israel, conducted on 3808 HCWs vaccinated with BNT162b2, demonstrated that the levels of IgG-specific Abs decreased at a consistent rate [14]. 

As previously described, the immune response after vaccination is likely driven by individual characteristics that include sex, age, various comorbidities, habits, etc. [39,40]. In our study, a significant difference in Ab levels was reported between two time points for both women and men, across all investigated vaccine groups. Moreover, this decline in Ab levels was also affected by the age of participants, with higher levels observed in younger groups especially in those vaccinated with BNT162b2 vaccine. “Immunosenescence” may contribute to the observed lower humoral response to vaccines in older age groups in our study, and that could be ascribed to intrinsic defects in B cells in the elderly population that include decreased class switch recombination and impaired memory B cell differentiation to plasma cells, as previously shown for influenza vaccination [41,42]. In line with our results, a large UK study with 45,965 adults from the general population, where Ab levels were measured at 3 months after receiving two doses of BNT162b2 or the ChAdOx1 vaccines, also demonstrated higher seropositivity to SARS-CoV-2 virus in younger age groups, in women, and those vaccinated with BNT162b2 in comparison to the ChAdOx1 vaccine [43]. On the other hand, Szebeni et al. investigated the effect of immunosuppressive treatment in patients with autoimmune rheumatic and musculoskeletal diseases on the production of anti-RBD neutralizing antibodies and on the SARS-CoV-2 specific T-cell response, based on the different SARS-CoV-2 vaccines. They found that, regardless of the risk factors that reduce immunogenicity in patients with autoimmune disease, the BBIBP-CorV vaccine appeared to produce the lowest, while BNT162b2 and mRNA-1273 vaccines produced the highest antibody response in healthy individuals and patients with autoimmune disease, both at one month and four months’ post immunization [44]. 

We also reported a strong correlation between measured Ab levels at two time points for all three observed vaccines, which may be contributing to the stable kinetics of Ab decline over time [20]. We further noticed the lowest mean %-change for Gam-COVID-Vac, followed by BNT162b2 and BBIBP-CorV vaccine, which may indicate different dynamics of waning of the immune response over time regarding application of different vaccines. This phenomenon might be affected by individual discrepancies but also by the characteristics of the vaccine type itself (adenovirus vector vaccine, mRNA, or inactivated whole-virus vaccine). A Japanese study, conducted in individuals vaccinated with BNT162b2, found a median %-change, from 3 to 6 months, to be −29.4%, with −31.6% in women and −25.1% in men, and authors of this study concluded that participants with initially lower Ab titers showed greater attenuation [34]. Results of the study from Israel, among 2653 participants, where Ab titer decay following BNT162b2 vaccination was measured, showed that the mean IgG titer decreased by 93.7% at 6 months, i.e., by up to 38% in each subsequent month [38]. In our study, we also reported a significant positive correlation between the %-change and the Ab levels 6 months after vaccination with BNT162b2, but also BBIBP-CorV and Gam-COVID-Vac. Considering these different dynamics of changes noticed by other authors, as well as in our study, a different vaccination schedule for the third dose across different vaccine types could be foreseen. On the other hand, we found no statistically significant differences between the Ab %-change regarding sex and age groups of participants for the investigated vaccine groups. Even though all vaccines induced a robust Ab response in the initial period this decreasing trend of Ab titers/levels might have been anticipated due to the fact that plasmablasts induced by vaccination do not necessarily differentiate into long-lived plasma cells [45,46]. 

In this study, we directly compared Ab levels at 6 months after administration of the second dose in four different vaccine groups matched by sex and age category. As a result, we found the highest mean Ab values for Gam-COVID-Vac, followed by BNT162b2, ChAdOx1 nCoV-19 and BBIBP-CorV. Participants vaccinated with Gam-COVID-Vac or with BNT162b2 had significantly higher values of Ab levels than those vaccinated with ChAdOx1 nCoV-19 and BBIBP-CorV. We also found that, 6 months after vaccination, individuals vaccinated with ChAdOx1 nCoV-19 had significantly higher Ab levels compared to those who received BBIBP-CorV vaccine. Results of the study conducted among 196 Mongolian fully vaccinated participants with one of the same four COVID-19 vaccines as in our study (BNT162b2, ChAdOx1 nCoV-19, Gam-COVID-Vac, and BBIBP-CorV), with a median sampling time between 2 and 3 months after the second dose, showed marked differences in Ab levels, with low Ab titers and RBD-ACE2 blocking activity after vaccination with BBIBP-CorV and Gam-COVID-Vac vaccines in comparison to the ChAdOx1 nCoV-19 or BNT162b2 vaccines [47]. These findings are not completely in line with ours, probably due to different time periods of observation and a later measurement of Ab levels in our study. A similar study conducted in Belarus, compared the immunogenicity and reactogenicity of Gam-COVID-Vac and BBIBP-CorV in 60 adults coming from the same population showing that the Gam-COVID-Vac vaccine was more immunogenic, and the BBIBP-CorV vaccine was less reactogenic [48]. Several studies investigated the immune response after application of the BNT162b2 vaccine as one of the widely administered SARS-CoV-2 vaccines worldwide, and even though high efficacy was announced after two doses of above 90% up to 6 months [49], an important long-term decline in Ab titers/levels was reported across several studies [14,19,20,38,50]. On the other hand, there are studies showing that even 8 months after the second dose of the BNT162b2, the total Ab titers are above the limit of detection [34].

In our study, among participants of the same vaccine group, there was no significant difference in detectable Ab levels regarding sex. However, there were noticeable differences between the BBIBP-CorV and each of the other investigated vaccines, in both women and men. A few studies, mostly conducted on BNT162b2, demonstrated higher Ab levels in women [32,51], although other authors found no sex-based differences [52]. An Italian study evaluated anti S-RBD IgG levels in 2248 vaccinated subjects and reported higher initial levels in women in comparison to men, although at 72 days after the first measurement this difference was lost [53]. Similarly, a study conducted in Brazil among HCWs vaccinated with inactivated vaccine (CoronaVac–Sinovac) did not find any difference measured at 6 months or even more, between different age groups and sexes [36]. On the other hand, a recent meta-analysis investigated the efficacy of BNT162b2, Gam-COVID-Vac, mRNA-1273-Moderna and Ad26.COV2.S-Johnson&Johnson/Janssen, showed a significantly increased efficacy in men compared to women [54]. Finally, the reported significant differences between BBIBP-CorV and each of the other investigated vaccines in our study might reflect generally lower levels of Abs measured in all vaccinated subjects with the BBIBP-CorV rather than a true effect of sex on Ab production. 

As for the age, we found no significant difference in Ab levels across the investigated age groups within the same vaccine group, except in those vaccinated with BNT162b2, where younger categories had mostly higher mean Ab levels in respect to the older. In the study by Fodor et al., in which humoral and cellular immune responses of five vaccines were compared, including the ChAdOx1, BNT162b2, mRNA-1273, BBIBP-CorV, and Gam-COVID-Vac vaccines, the protection obtained after the administration of mRNA-based vaccines was more robust than the one after other vaccines by promoting a significantly higher T-cell response as well as higher levels of anti-spike IgG and neutralizing antibodies, regardless of the age and sex of participants [55]. The SARS-CoV-2 mRNA vaccines, including BNT162b2, were able to elicit a robust and persistent T follicular helper cell response in humans [56]. In the aged individuals, the immune response after vaccination mostly favored the short-lived effector T cells over the long-term memory precursors and follicular helper T cells, which might explain our findings [57]. Increasing age of the participants was found to modestly correlate with decreasing Ab levels at 6 months after application of the second dose of BNT162b2 and ChAdOx1 nCoV-19 in our study. Similar findings were reported in participants where Ab levels 6 months after vaccination with BNT162b2 decreased more rapidly in older individuals [58]. Contrary to this, another research conducted in a smaller group of participants revealed no association between the Ab levels and the age in naïve vaccinees even at 120 days after vaccination with BNT162b2 [59].

As mentioned before, the protective Ab titers/levels are not yet precisely defined [4,10,54]. Thus, only the continuous surveillance of the population can help to clarify aspects of the VE in terms of the duration of protection and the need for booster doses of the previously vaccinated persons [4]. Even though this issue was beyond the scope of the current study, we found that the majority of recipients vaccinated with BNT162b2, Gam-COVID-Vac and ChAdOx1 nCoV-19 remained seropositive to SARS-CoV-2 virus at 6 months, in comparison to 40% of those vaccinated with BBIBP-CorV, with no significant differences regarding sex. A lower percentage of seropositivity at 6 months after vaccination with another inactivated SARS-CoV-2 vaccine (CoronaVac) was previously demonstrated in a similar study by Taminato et al., where only 20% of participants were seropositive for more than 6 months [36]. Of note, measuring just the Ab response against S protein, especially after being vaccinated with the whole-virus vaccines, such as BBIBP-CorV, might not be enough to evaluate the complete immune response, since the production of other Abs against different SARS-CoV-2 antigens is also induced [23]. In this situation, a study that evaluates the VE is crucial, as we previously described in detail [22].

Our study has strengths and several limitations that should be acknowledged. This is the first study in our region, and one of the few studies that systematically evaluates the Ab response 6 months after administration of one of four different SARS-CoV-2 vaccines that include rarely studied Gam-COVID-Vac and BBIBP-CorV vaccines in two time-point measurements. In addition, we carefully selected eligible individuals covering both sexes and a wide age range. Importantly, our results were robust to sensitivity analyses. Limitations, on the other hand, are the lack of information on potential medications used and/or presence of chronic diseases that may have had a potential impact on participants’ immune response. Second, there is a lack of data on Ab levels prior to vaccination. More precisely, even though we considered only vaccinated individuals who did not have previous positive tests for SARS-CoV-2 (either by PCR or RDT-Ag) and also excluded participants who became infected in the period between two measurements, we cannot completely exclude the possibility of an asymptomatic infection before enrollment, which may have influenced the measured Ab values. However, we believe that this limitation did not have a significant impact on the main results and the conclusions of this study. Third, we did not measure antibodies targeting the viral N protein (which is contained in the whole virus inactivated vaccine), which would enable a differentiation between a vaccine-induced response and a response induced by natural exposure to SARS-CoV-2 in those vaccinated with BNT162b2 or Gam-COVID-Vac vaccine (but not those vaccinated with BBIBP-CorV), thus our results should be interpreted with caution. We primarily focused on measuring the Ab response to spike protein after the vaccination as an important component of immunity to SARS-CoV-2, even though the T cell response and memory B cells are also very important for the long-term protection against viral infections like COVID-19 [60,61]. Although a reduction of measured Ab levels does not necessarily imply a reduction in protection against SARS-CoV-2 [62], testing of cell-mediated immunity was beyond the scope of the current study. Fourth, the lack of virus neutralization assays also adds to the limitations of our study. Of note was a high correlation between the Ab response to the spike protein that we analyzed, and pseudovirus neutralization demonstrated in previous research [63,64]. Another limitation was the measuring range interval of the used assay (3.8–400.0 AU/mL) which was unable to precisely determine the Ab concentration below and above this limit of quantitation, but we conducted a sensitivity statistical analysis by limiting our sample to just those participants with measured Ab levels below 400.0 AU/mL in order to explore the effect of this issue on the overall results. In conclusion, our results were robust to several sensitivity analyses, allowing us to conclude that this limitation had a negligible impact on our initial findings. Sixth, even though the number of participants was relatively small for additional stratified analyses, since many of the potentially eligible participants already received a third dose before arriving at 6 months, our study still provided some valuable findings. In addition, we were able to follow the majority of our participants longitudinally at two time points. Seventh, a cause-effect relationship for the observed associations was not possible to determine due to the observational nature of this study. Even though beyond the aim of our study, we cannot conclude how well these measured levels of Abs after vaccination correlate with the protection against Omicron variants (e.g., BA.5), nor against some new variants of concern of SARS-CoV-2 that might emerge in the near future. However, a recent study by Gerges et al. [65] concluded that it can be assumed that a small amount of IgG cannot neutralize the Omicron variant, although the higher titers of those antibodies may express some level of neutralizing activity, contributing to a milder clinical presentation of disease in these individuals by cross-recognition of the S-protein RBD between the variants and broadening the adaptive immunity [66].

## 5. Conclusions

This longitudinal follow-up evaluation demonstrated a decline in the levels of anti-spike IgG to SARS-CoV-2 virus at 6 months post-vaccination in participants vaccinated with BNT162b2, BBIBP-CorV, and Gam-COVID-Vac, but the seropositivity to SARS-CoV-2 virus remained at above 85% for those vaccinated with BNT162b2 or Gam-COVID-Vac, although for those who received BBIBP-CorV, it was below 40%. After matching the participants, vaccinated with different vaccines, we found a significant difference in Ab levels at 6 months following the application of the second dose of vaccine between those vaccinated with either of the three vaccines (BNT162b2, Gam-COVID-Vac, and ChAdOx1 nCoV-19) and BBIBP-CorV, as well as between those vaccinated with BNT162b2 and ChAdOx1 nCoV-19. In comparison with the older age categories, younger participants had a higher mean Ab level when they were vaccinated with BNT162b2. In the absence of VE estimation, these results potentially suggest a lower protection against SARS-CoV-2 infection over time, especially in those vaccinated with BBIBP-CorV. 

In order to obtain the optimal public health benefit, a booster with the third dose 6 months after the second dose became available in Serbia, in August 2021, when Delta variant of SARS-CoV-2 was predominant, and recently also a fourth dose was recommended for high-risk groups, HCWs and older population [21]. Findings from our study support these decisions, in particular for those initially vaccinated with the BBIBP-CorV vaccine, in whom subsequent administration of RNA vaccine, like BNT162b2, would provide much stronger humoral response and presumably better protection [67]. Additionally, our results can be of importance for the public health decisions in the countries where vaccination is still in the early phase or in consideration for implementation of the additional booster dose. Also, as a proof of response to the immunization, it may contribute to a positive public perception of COVID-19 vaccination and as such may have a positive impact in combating skepticism and rumors towards the SARS-CoV-2 vaccines. Finally, further large-scale studies are warranted to determine whether the SARS-CoV-2 specific Ab decline continues in the following time points or it reaches a plateau, as well as, to better understand the relationship between the Ab decline and vaccine’s efficacy/effectiveness. 

## Figures and Tables

**Table 1:** Antibody levels on 28 days and 6 months after administration of the second dose of vaccine, stratified by sex and age groups of participants.

Vaccine	At the 28th Day after the Second Dose							At 6 Months after the Second Dose					
	*n*	%	Mean (AU/mL)	SD	Median (AU/mL)	IQR (25–75)		Mean (AU/mL)	SD	Median (AU/mL)	IQR (25–75)		*p*-Value ^1^
**BNT162b2**													
**Total**	130	100	216.55	105.7	209	135	288	75.67	57.3	67.95	37.3	101	**<0.001**
**Sex**													
Female	75	57.7	219.24	102.2	210	140	297	76.54	49.24	71.6	37.3	103	**<0.001**
Male	55	42.3	212.88	111.3	204	121	280	74.48	67.23	56.9	33.5	95.2	**<0.001**
**Age category**													
20–29	5	3.85	255.4	93.92	254	238	259	90.9	34.18	77.7	67.2	105	0.063
30–39	12	9.23	276.46	108	288.5	198.5	392	117.28	88.45	92.5	56.1	151	**0.002**
40–49	41	31.5	217.8	114.6	199	135	306	76.45	65.31	70.9	36	98.2	**<0.001**
50–59	35	26.9	222.21	82.61	212	157	288	75.12	41.68	68.7	47.7	101	**<0.001**
60–69	15	11.5	255.24	125.2	239	133	401	88.08	50.57	83.8	60.5	121	**<0.001**
≥70	22	16.9	137.32	65.34	131	97.8	199	40.47	27.48	35.8	12.7	60.6	**<0.001**
**BBIBP-CorV**													
**Total**	53	100	72.74	80.04	48.1	22.7	78.6	24.43	38.43	8.13	4.37	21.9	**<0.001**
**Sex**													
Female	34	64.2	72.29	87.2	44.7	22.3	72.7	24.94	44.4	7.61	4.37	21.4	**<0.001**
Male	19	35.9	73.55	67.55	51.8	22.7	95.2	23.52	25.52	10	3.79	38.2	**<0.001**
**Age category**													
20–29	1	1.89	230		230			94		94			NA
30–39	9	17	120.89	132.7	55.1	40.3	118	56.95	74.4	8.48	5.57	97.1	**0.004**
40–49	18	34	50.87	46.57	46.1	13.9	69.7	16.08	18.19	7.98	4.22	20.7	**<0.001**
50–59	16	30.2	73.39	72.96	46.4	23.5	84	19.05	24.01	7.61	4.39	24.6	**<0.001**
60–69	4	7.55	43.37	38.17	35.1	13.99	72.8	12.37	10.06	11.3	3.79	20.95	0.125
≥70	5	9.43	54.74	54.59	51.1	13.1	51.6	8.94	4.99	10	4.23	11	0.063
**Gam-COVID-Vac**													
**Total**	48	100	194.38	140.2	147	85.8	401	90.53	111.3	36.3	19.75	112	**<0.001**
**Sex**													
Female	31	64.6	176.38	139.4	111	78.2	369	81.44	113.8	28	17	97.9	**<0.001**
Male	17	35.4	227.21	140	189	132	401	107.11	108.1	82.1	36.7	124	**<0.001**
**Age category**													
20–29	1	2.08	154		154			83.7		83.7			NA
30–39	5	10.4	151.64	51.38	162	132	168	46.96	35.09	42.6	34.1	45.7	0.063
40–49	17	35.4	201.02	158.5	140	60.8	401	114.36	138.6	45.5	21	105	**<0.001**
50–59	14	29.2	257.58	148.4	284	96.6	401	117.51	117.6	100.55	22.6	186	**<0.001**
60–69	7	14.6	138.71	129.5	103	38.1	195	49.11	71.89	35.9	6.18	43.1	**0.016**
≥70	4	8.33	105.9	39.49	103.2	74.3	138	23.48	6.31	23.6	18.1	28.85	0.125

Notes: For statistical processing and presentation of data, results below the minimum detectable value of the assay (<3.8 AU/mL) were interpreted as 3.79, and above the maximum detectable value (>400 AU/mL) as 401. ^1^ Wilcoxon matched-pairs signed-rank test (exact test, where applicable), *p*-value refers to the difference between matched groups at two time points of measurements within the same vaccine. *p*-values that are statistically significant at *p* < 0.05 are presented in bold. SD = standard deviation, IQR = interquartile range, NA = not applicable.

**Table 3:** Antibody levels at 6 months from the administration of the second dose of vaccine, stratified by sex and age of participants.

	BNT162b2 Vaccine				BBIBP-CorV Vaccine				Gam-COVID-Vac Vaccine				ChAdOx1 nCoV-19 Vaccine				
	*n* (%)	Mean (SD), AU/mL	Median (IQR, 25–75), AU/mL	*p*-Value ^1^	*n* (%)	Mean (SD), AU/mL	Median (IQR, 25–75), AU/mL	*p*-Value ^1^	*n* (%)	Mean (SD), AU/mL	Median (IQR, 25–75), AU/mL	*p*-Value ^1^	*n* (%)	Mean (SD), AU/mL	Median (IQR, 25–75), AU/mL	*p*-Value ^1^	*p*-Value ^2^
**Total**	70 (100)	85.25 (60.02)	74.55 (42.70–109.00)	NA	70 (100)	25.26 (36.92)	8.18 (4.37–27.40)	NA	70 (100)	91.28 (95.96)	44.85 (25.00–127.00)	NA	70 (100)	64.22 (65.30)	50.60 (19.00–93.20)	NA	**<0.01**
**Sex**																	
Female	35 (50.00)	81.47 (47.87)	77.70 (43.80–103.00)	0.91	35 (50.00)	24.86 (42.68)	7.59 (4.22–22.40)	0.52	35 (50.00)	90.44 (103.47)	42.70 (20.30–137.00)	0.47	35 (50.00)	68.12 (69.11)	52.40 (29.20–97.70)	0.47	**<0.01**
Male	35 (50.00)	89.03 (70.64)	70.90 (38.20–123.00)		35 (50.00)	25.65 (30.72)	9.95 (5.20–41.60)		35 (50.00)	92.11 (89.33)	57.90 (29.70–124.00)		35 (50.00)	60.32 (62.00)	41.70 (14.50–93.20)		**<0.01**
**Age category**																	
20–29	6 (8.57)	145.30 (79.72)	130.75 (77.70–230.00)	**<0.01**	6 (8.57)	28.44 (36.64)	7.45 (7.04–50.50)	0.69	8 (11.43)	100.65 (61.23)	110.35 (46.00–138.00)	0.45	10 (14.29)	118.10 (133.92)	74.35 (21.10–128.00)	0.61	0.06
30–39	12 (17.15)	113.06 (88.31)	92.50 (54.20–147.50)		12 (17.15)	40.99 (64.81)	8.47 (5.65–51.05)		12 (17.15)	79.95 (65.90)	63.40 (38.35–105.50)		14 (20.00)	53.44 (22.88)	44.60 (36.10–78.30)		**0.01**
40–49	18 (25.71)	74.38 (35.68)	76.70 (40.40–98.20)		18 (25.71)	18.37 (20.67)	7.98 (4.22–21.90)		18 (25.71)	93.58 (119.76)	34.05 (16.60–105.00)		12 (17.14)	61.16 (47.13)	38.35 (22.95–106.50)		**<0.01**
50–59	12 (17.15)	56.56 (35.50)	52.40 (35.75–79.00)		14 (20.00)	24.86 (26.81)	9.54 (4.40–38.20)		12 (17.15)	118.89 (113.21)	100.55 (29.15–156.50)		14 (20.00)	64.87 (51.82)	67.20 (12.10–99.40)		**<0.01**
60–69	16 (22.85)	95.19 (45.83)	93.40 (67.25–122.00)		12 (17.14)	30.50 (40.08)	19.30 (3.79–42.10)		16 (22.85)	86.17 (100.15)	38.85 (20.30–166.50)		14 (20.00)	50.73 (37.65)	54.25 (15.20–63.30)		**<0.01**
≥70	6 (8.57)	33.02 (24.99)	30.65 (11.70–49.10)		8 (11.43)	7.58 (4.26)	6.64 (3.79–10.15)		4 (5.71)	33.73 (17.32)	30.00 (22.50–44.95)		6 (8.57)	35.67 (44.66)	14.60 (9.25–51.80)		**0.04**

Notes: For statistical processing and presentation of data, results below the minimum detectable value of the assay (<3.8) were interpreted as 3.79, and above the maximum detectable value (> 400) as 401. Statistically significant *p*-values are presented in bold. SD = standard deviation, IQR = interquartile range, NA = not applicable. ^1^ Wilcoxon rank-sum or Kruskal–Wallis test; *p*-value refers to difference between groups within the same vaccine. ^2^ Kruskal-Wallis test; *p*-value refers to difference between groups across different vaccines.

**Table 4:** Serological profile of the study participants at 6 months from the administration of the second dose of four different SARS-CoV-2 vaccines.

	Total	BNT162b2 Vaccine (*n* = 70)				BBIBP-CorV Vaccine (*n* = 70)				Gam-COVID-Vac Vaccine (*n* = 70)				ChAdOx1 nCoV-19 Vaccine (*n* = 70)			
		Sero-Negative	Equi-Vocal	Sero-Positive	*p*-Value ^1^	Sero-Negative	Equi-Vocal	Sero-Positive	*p*-Value ^1^	Sero-Negative	Equi-Vocal	Sero-Positive	*p*-Value ^1^	Sero-Negative	Equi-vocal	Sero-Positive	*p*-Value ^1^
**Total,** ***n*** **(%)**	280 (100)	4 (5.71)	0	66 (94.29)	NA	42 (60.00)	0	28 (40.00)	NA	10 (14.29)	0	60 (85.71)	NA	11 (15.71)	4 (5.72)	55 (78.57)	NA
**Sex, *n* (%)**																	
Female	140 (100)	2 (5.71)	0	33 (94.29)	1	22 (62.86)	0	13 (37.14)	0.81	5 (14.29)	0	30 (85.71)	1	4 (11.43)	2 (5.71)	29 (82.86)	0.69
Male	140 (100)	2 (5.71)	0	33 (94.29)		20 (57.14)	0	15 (42.86)		5 (14.29)	0	30 (85.71)		7 (20.00)	2 (5.71)	26 (74.29)	
**Age (years), mean (SD)**	49.17 (14.34)	66.75 (14.68)	NA	48.88 (14.68)	**0.02**	50.26 (15.60)	NA	49.54 (13.86)	0.84	52.30 (9.06)	NA	47.90 (13.76)	0.33	54.00 (16.30)	55.25 (12.95)	46.62 (13.98)	0.8
**Age category, *n* (%)**																	
**20–29**	30 (100)	0	0	6 (100)	**<0.01**	4 (66.67)	0	2 (33.33)	0.51	0	0	8 (100)	0.76	2 (20.00)	0	8 (80.00)	0.16
**30–39**	50 (100)	0	0	12 (100)		7 (58.33)	0	5 (41.67)		1 (8.33)	0	11 (91.67)		0	0	14 (100)	
**40–49**	66 (100)	0	0	18 (100)		11 (61.11)	0	7 (38.89)		4 (22.22)	0	14 (77.78)		1 (8.33)	1 (8.33)	10 (83.34)	
**50–59**	52 (100)	2 (16.67)	0	10 (83.33)		8 (57.14)	0	6 (42.86)		2 (16.67)	0	10 (83.33)		3 (21.43)	2 (14.28)	9 (64.29)	
**60–69**	58 (100)	0	0	16 (100)		5 (41.67)	0	7 (58.33)		3 (18.75)	0	13 (81.25)		3 (21.43)	0	11 (78.57)	
**≥70**	24 (100)	2 (33.33)	0	4 (66.67)		7 (87.50)	0	1 (12.50)		0	0	4 (100)		2 (33.33)	1 (16.67)	3 (50.00)	

Notes: participants were classified based on the Ab levels at 6 months after vaccination as: seronegative (<12.0 AU/mL), equivocal (12.0–15.0 AU/mL), seropositive (>15.0 AU/mL). ^1^ Pearson’s chi-squared test (Fisher’s exact test) for categorical and t-test or ANOVA-analysis of variance for continuous variables. Significance levels are given in bold for *p* < 0.05. NA = not applicable, SD = standard deviation.

## Data Availability

The data that support the findings of this study are available from the corresponding author upon reasonable request.

## Supplementary Materials

The following supporting information can be downloaded at: https://www.mdpi.com/article/10.3390/vaccines11010056/s1, Figure S1: Proportion of seropositivity at the 28th day and 6 months after the second dose of vaccine, by sex (A) and age groups (B) of participants; Figure S2: Comparison of the percentage change in Ab levels between the 28th day and at 6 months from the administration of the second dose of vaccines, stratified by sex (A) and age groups (B) of participants; Figure S3: Correlation between the percentage change and Ab levels measured at the 28th day from the administration of the second dose of BNT162b2 (A), BBIBP-CorV (B) and Gam-COVID-Vac (C) vaccines; Figure S4: Correlation between the percentage change in Ab levels and levels at 6 months from the administration of the second dose of BNT162b2 (A), BBIBP-CorV (B) and Gam-COVID-Vac (C) vaccines; Figure S5: Correlation between the percentage change in Ab levels and age of participants, by BNT162b2 (A), BBIBP-CorV (B) and Gam-COVID-Vac (C) vaccines; Figure S6: Correlation between antibody levels measured 6 months post-vaccination after the second dose of BNT162b2 (A), BBIBP-CorV (B), Gam-COVID-Vac (C) and ChAdOx1 nCoV-19 (D) vaccines, and the age of participants. Supplementary File S1: Results of the sensitivity analysis.
