# Supplementary material for: Immunogenicity of BNT162b2, BBIBP-CorV, Gam-COVID-Vac and ChAdOx1 nCoV-19 Vaccines Six Months after the Second Dose: A Longitudinal Prospective Study
Source: Vaccines (Basel). 2022 Dec 26;11(1):56. doi: 10.3390/vaccines11010056 (PMC9865554; doi:10.3390/vaccines11010056)
Supplement: Supplementary file 1 [file vaccines-11-00056-s001.zip › Supplementary File S1.pdf]

Supplementary file 1. Results of the sensitivity analysis

Table of Contents

Supplementary Note Fig 1. Distribution of participants, stratified by vaccine type, sex and number of samples (single or paired), after exclusion of one participant with Ab level above the test's upper limit of quantitation (>400 AU/mL) and three age- and sex- matching participants……..……….…….…………3

Supplementary Note Fig 2. Antibody levels at 28th day and 6 months after the second dose of vaccine, stratified by sex (A) and age groups (B) of participants, after exclusion of one participant with Ab level above the test's upper limit of quantitation (>400 AU/mL) and three age- and sex- matching participants…………………………………………………………………………………………..………..4

Supplementary Note Fig 3. Correlation between Ab levels at 28th day and 6 months after the second dose of BNT162b2 (A), BBIBP-CorV (B) and Gam-COVID-Vac (C) vaccines, after exclusion of one participant with Ab level above the test's upper limit of quantitation (>400 AU/mL) and three age- and sex- matching participants……………...…………………………………………………………………….……...……….5

Supplementary Note Fig 4. Antibody levels at 6 months after the administration of BNT162b2, BBIBP-CorV, Gam-COVID-Vac and ChAdOx1 nCoV-19 vaccines, stratified by sex (A) and age groups (B) of participants, after exclusion of one participant with Ab level above the test's upper limit of quantitation (>400 AU/mL) and three age- and sex- matching participants..…………………………………………....6

Supplementary Note Fig S1. Proportion of seropositive participants at 28th day and 6 months after the second dose of vaccine, by sex (A) and age groups (B) of participants, after exclusion of the participants with Ab levels above the test's upper limit of quantitation (>400 AU/mL)….......................…………………7

Supplementary Note Fig S2. Comparison of the percent change in Ab levels between 28th day and at 6 months from the administration of the second dose of vaccines, stratified by sex (A) and age groups (B) of participants, after exclusion of the participants with Ab levels above the test's upper limit of quantitation (>400 AU/mL) ……………………………………………………………………………….…….……….8

Supplementary Note Fig S3. Correlation between %-change and Ab levels measured at 28th day from the administration of the second dose of BNT162b2 (A), BBIBP-CorV (B) and Gam-COVID-Vac (C) vaccines, after exclusion of the participants with Ab levels above the test's upper limit of quantitation (>400 AU/mL)……………………………...…………………………………………………………………...…9

Supplementary Note Fig S4. Correlation between percent change in Ab levels and levels at 6 months from the administration of the second dose of BNT162b2 (A), BBIBP-CorV (B) and Gam-COVID-Vac (C) vaccines, after exclusion of the participants with Ab levels above the test's upper limit of quantitation (>400 AU/mL)..…………………..……………………………………………………………….….….…….10

Supplementary Note Fig S5. Correlation between percent change in Ab levels and age of participants, by BNT162b2 (A), BBIBP-CorV (B) and Gam-COVID-Vac (C) vaccines, after exclusion of the participants with Ab levels above the test's upper limit of quantitation (>400 AU/mL)………………..….….……………11

Supplementary Note Fig S6. Correlation between antibody levels measured 6 months post-vaccination after the second dose of BNT162b2 (A), BBIBP-CorV (B), Gam-COVID-Vac (C) and ChAdOx1 nCoV-19

(D) vaccines, and the age of participants, after exclusion of the participants with Ab levels above the test's upper limit of quantitation (>400 AU/mL) ……………………………………………………………..…..…….12

Supplementary Note Table 1. Antibody levels on 28th day and 6 months after administration of the second dose of vaccine, stratified by sex and age groups of the participants, after exclusion of the participants with Ab levels above the test's upper limit of quantitation (>400 AU/mL)…………..……….13

Supplementary Note Table 2. Percent change in Ab levels between 28th day and at 6 months, stratified by sex and age groups, after exclusion of the participants with Ab levels above the test's upper limit of quantitation (>400 AU/mL)………..…………………………………………….……….…….……..…….15

Supplementary Note Table 3. Antibody levels at 6 months from the administration of the second dose of vaccine, stratified by sex and age of the participants, after exclusion of one participant with Ab level above the test's upper limit of quantitation (>400 AU/mL) and three age- and sex- matching participants…………………...…………………………………………………………….…….……….16

Supplementary Note Table 4. Serological profile of the study participants at 6 months from the administration of the second dose of four different SARS-CoV-2 vaccines, after exclusion of one participant with Ab level above the test's upper limit of quantitation (>400 AU/mL) and three age- and sex- matching participants…………………………………………………………………….…..………..18

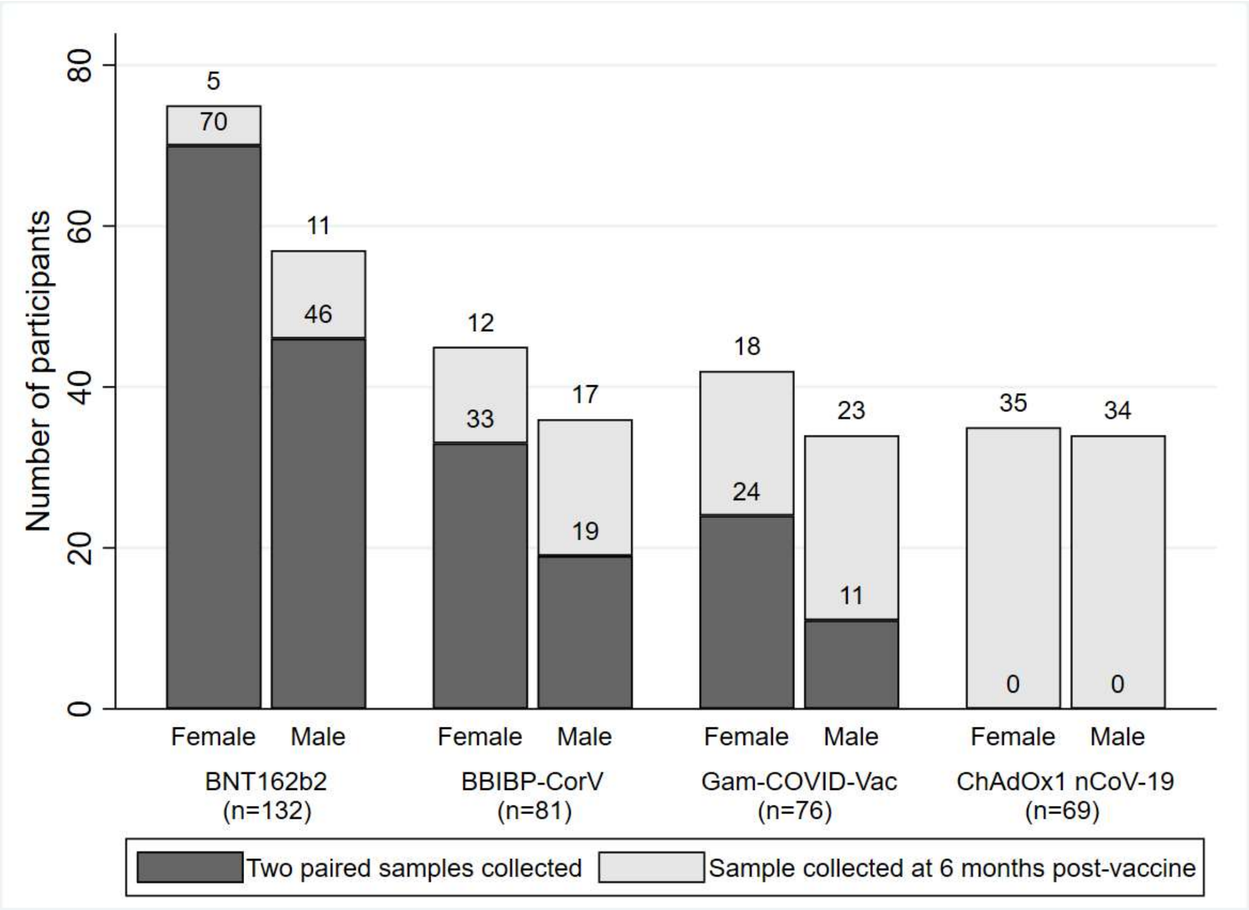

Supplementary Note Fig 1. Distribution of participants, stratified by vaccine type, sex and number of samples (single or paired), after exclusion of one participant with Ab level above the test's upper limit of quantitation (>400 AU/mL) and three age- and sex- matching participants.

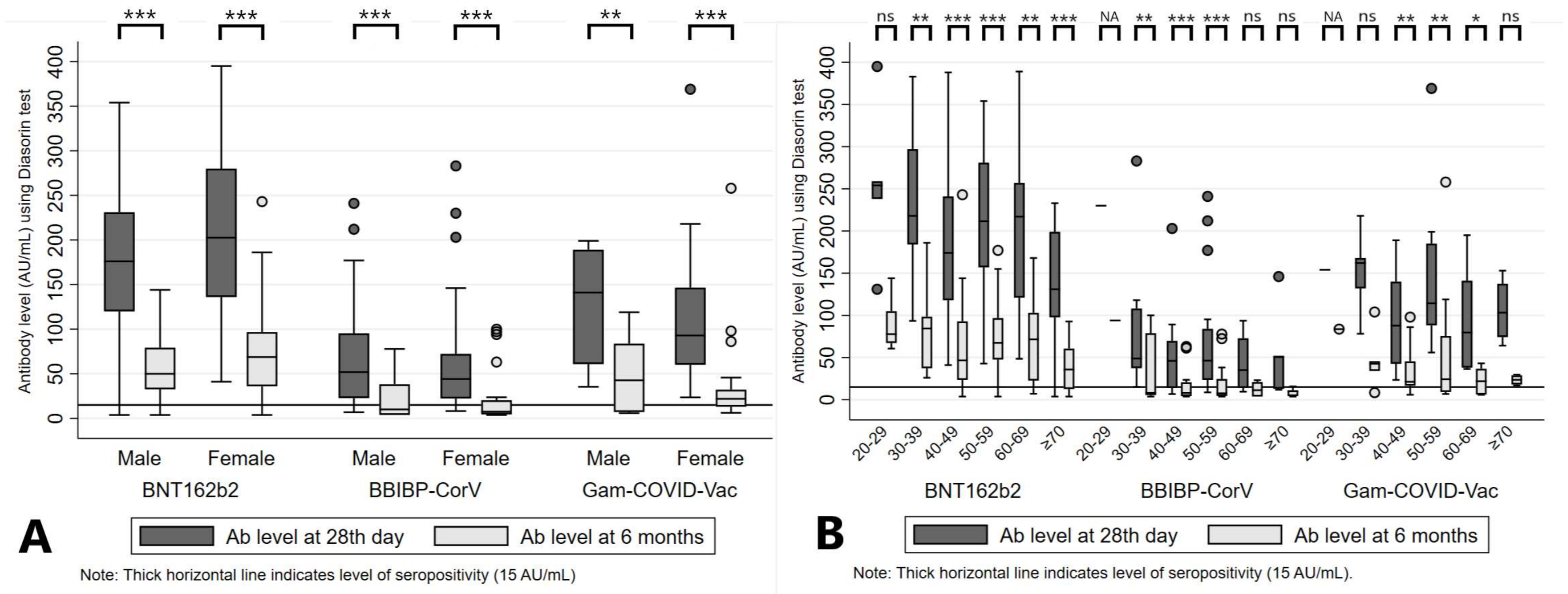

Supplementary Note Fig 2. Antibody levels at 28th day and 6 months after the second dose of vaccine, stratified by sex (A) and age groups (B) of participants, after exclusion of one participant with Ab level above the test's upper limit of quantitation (>400 AU/mL) and three age- and sex-matching participants ***p<0.001, **p<0.01, *p<0.05, ns = not significant.

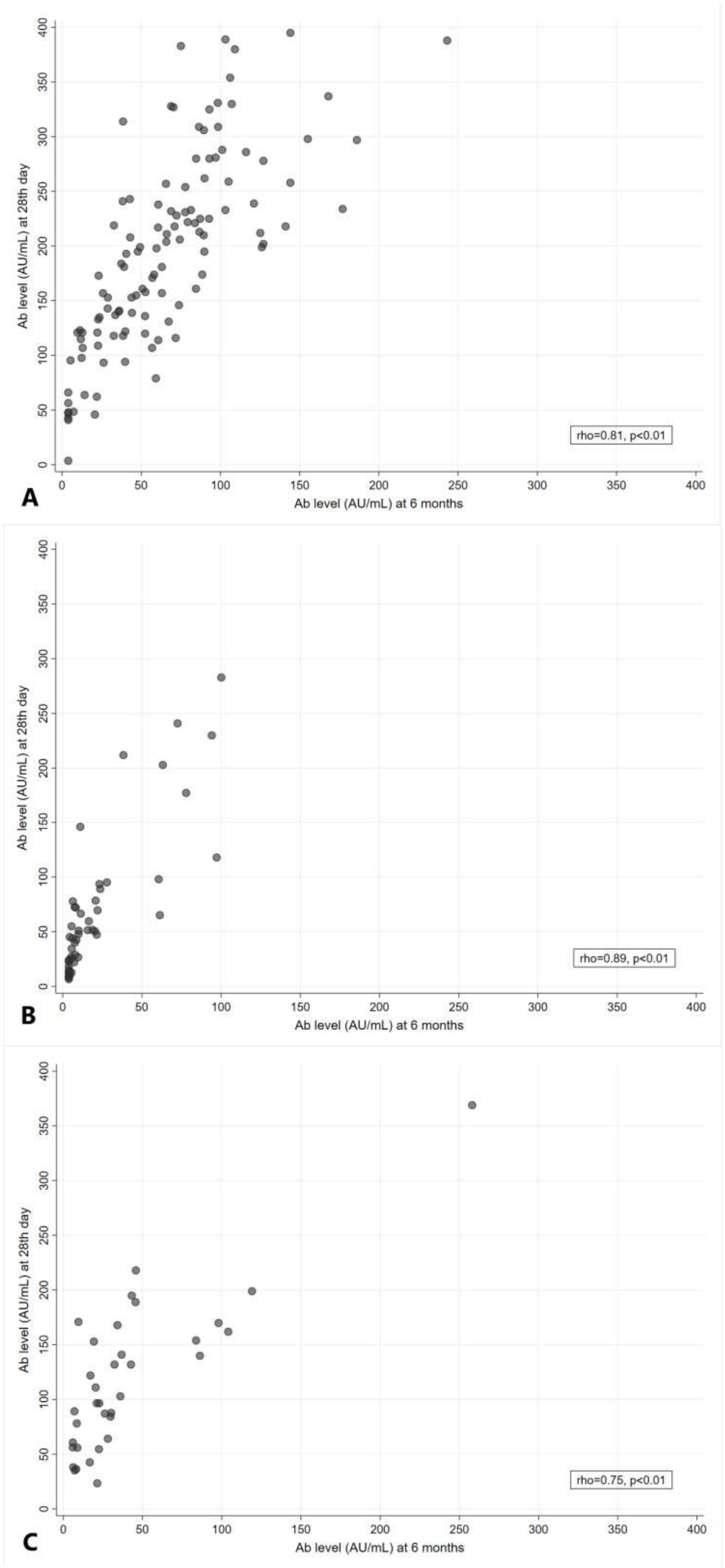

Supplementary Note Fig 3. Correlation between Ab levels at 28th day and 6 months after the second dose of BNT162b2 (A), BBIBP-CorV (B) and Gam-COVID-Vac (C) vaccines, after exclusion of one participant with Ab level above the test's upper limit of quantitation (>400 AU/mL) and three age- and sex- matching participants. rho = Spearman's correlation coefficient.

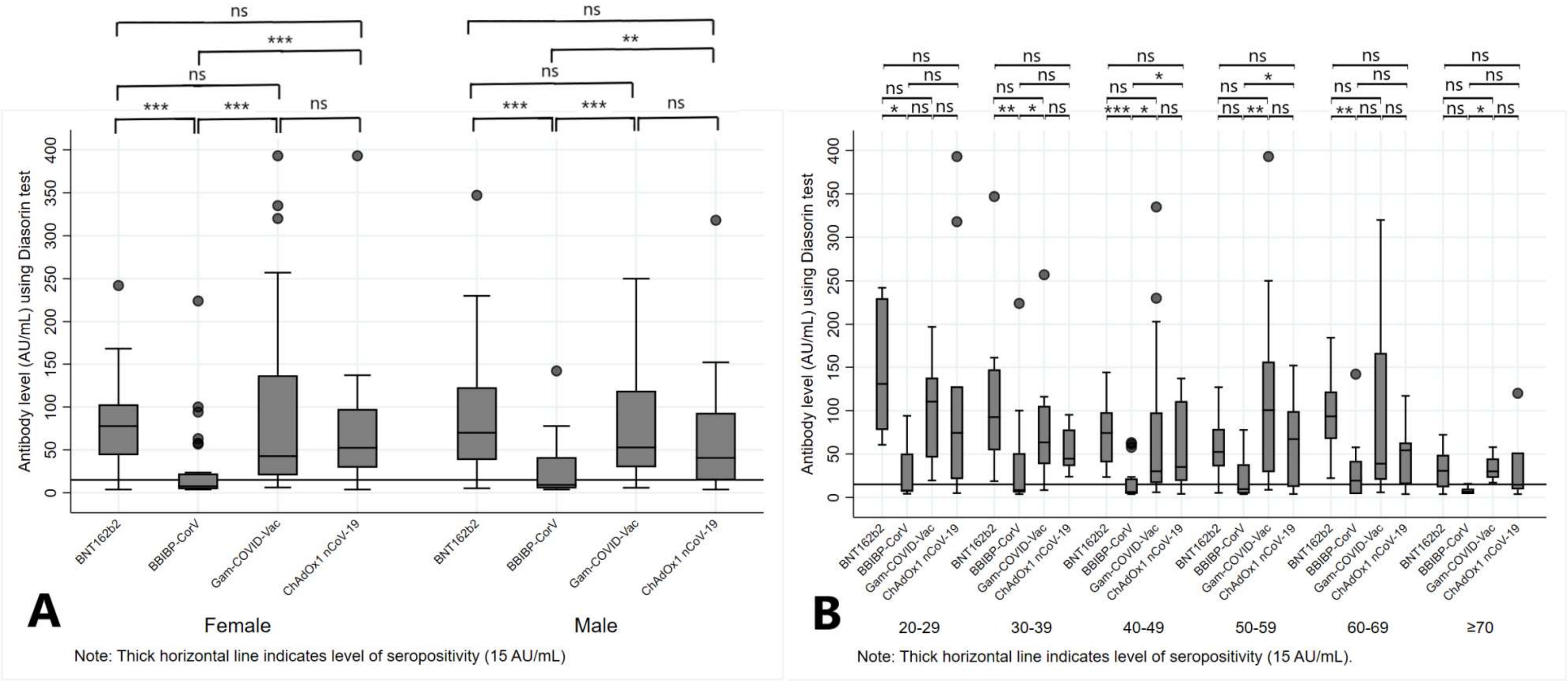

Supplementary Note Fig 4. Antibody levels at 6 months after the administration of BNT162b2, BBIBP-CorV, Gam-COVID-Vac and ChAdOx1 nCoV-19 vaccines, stratified by sex (A) and age groups (B) of participants, after exclusion of one participant with Ab level above the test's upper limit of quantitation (>400 AU/mL) and three age- and sex- matching participants. Legend: ***p<0.001, **p<0.01, *p<0.05, ns = not significant.

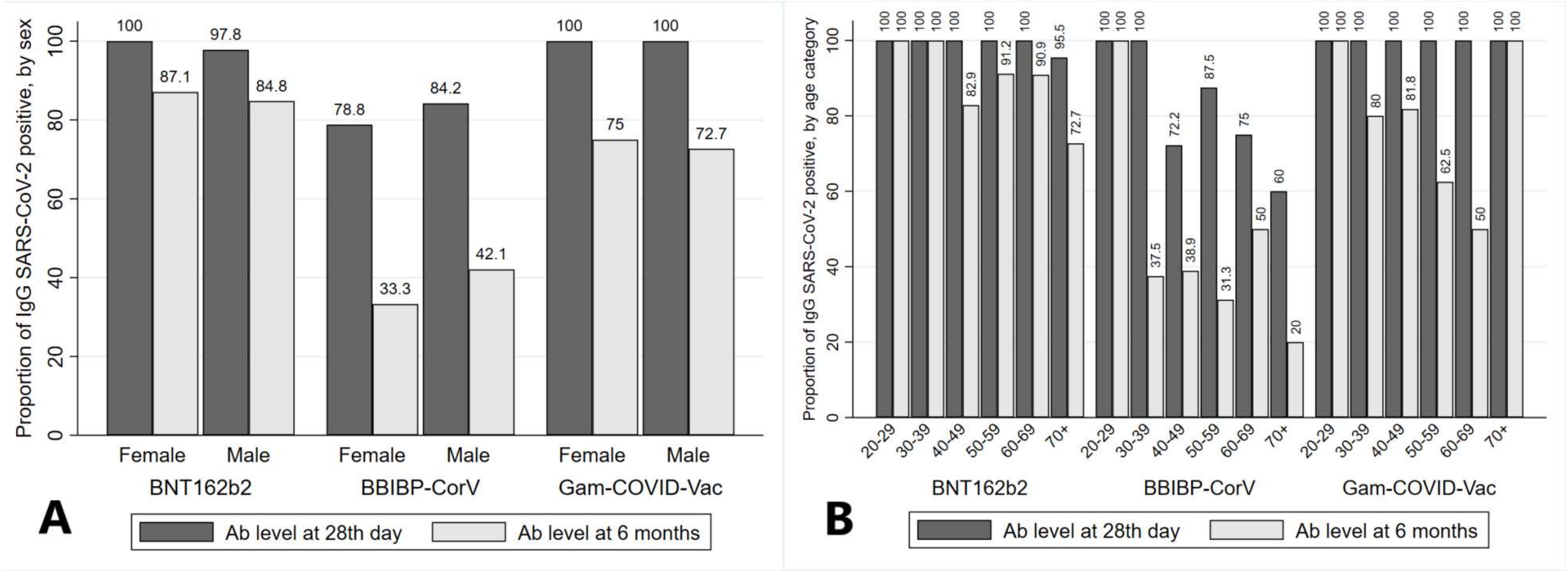

Supplementary Note Fig S1. Proportion of seropositive participants at 28th day and 6 months after the second dose of vaccine, by sex (A) and age groups (B) of participants, after exclusion of the participants with Ab levels above the test's upper limit of quantitation (>400 AU/mL).

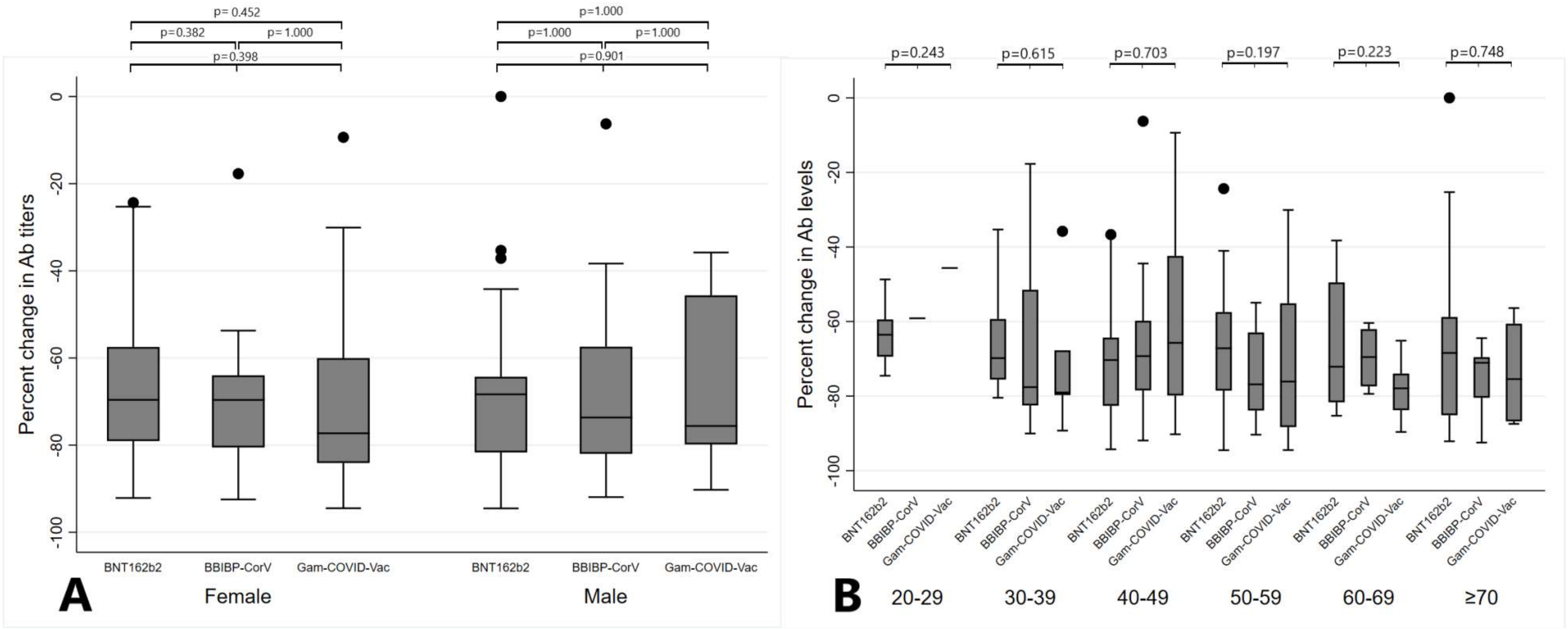

Supplementary Note Fig S2. Comparison of the percent change in Ab levels between 28th day and at 6 months from the administration of the second dose of vaccines, stratified by sex (A) and age groups (B) of participants, after exclusion of the participants with Ab levels above the test's upper limit of quantitation (>400 AU/mL).

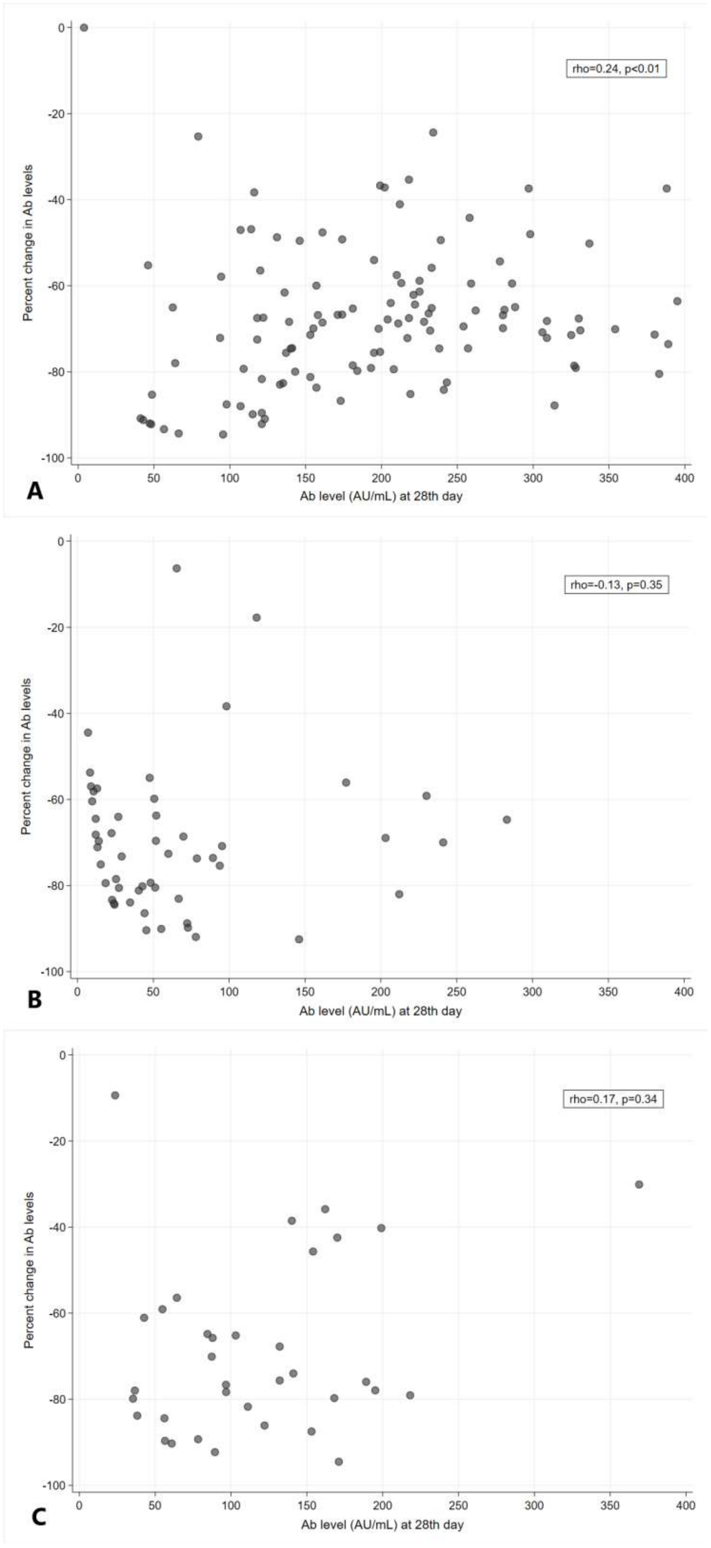

Supplementary Note Fig S3. Correlation between %-change and Ab levels measured at 28th day from the administration of the second dose of BNT162b2 (A), BBIBP-CorV (B) and Gam-COVID-Vac (C) vaccines, after exclusion of the participants with Ab levels above the test's upper limit of quantitation (>400 AU/mL). rho = Spearman's correlation coefficient.

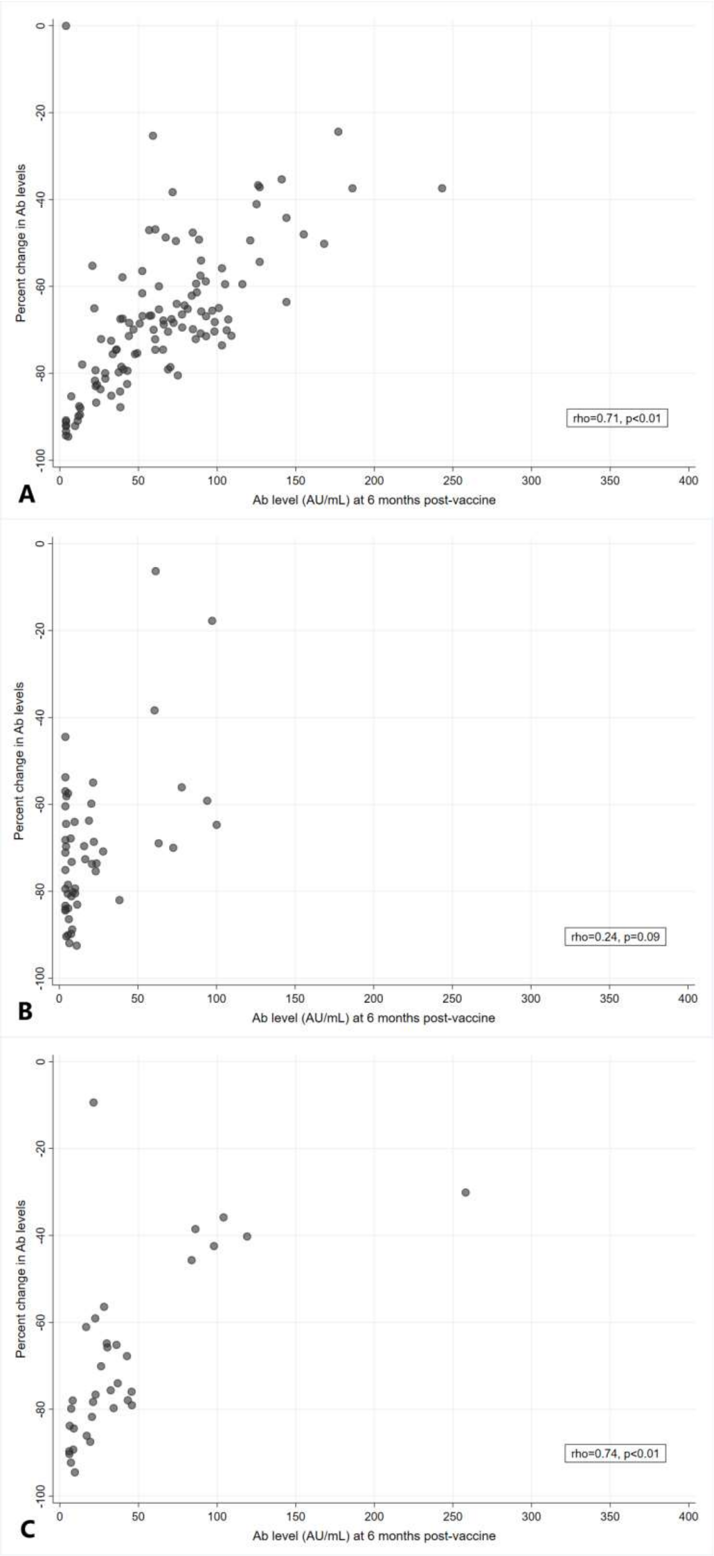

Supplementary Note Fig S4. Correlation between percent change in Ab levels and levels at 6 months from the administration of the second dose of BNT162b2 (A), BBIBP-CorV (B) and Gam-COVID-Vac (C) vaccines, after exclusion of the participants with Ab levels above the test's upper limit of quantitation (>400 AU/mL). rho = Spearman's correlation coefficient.

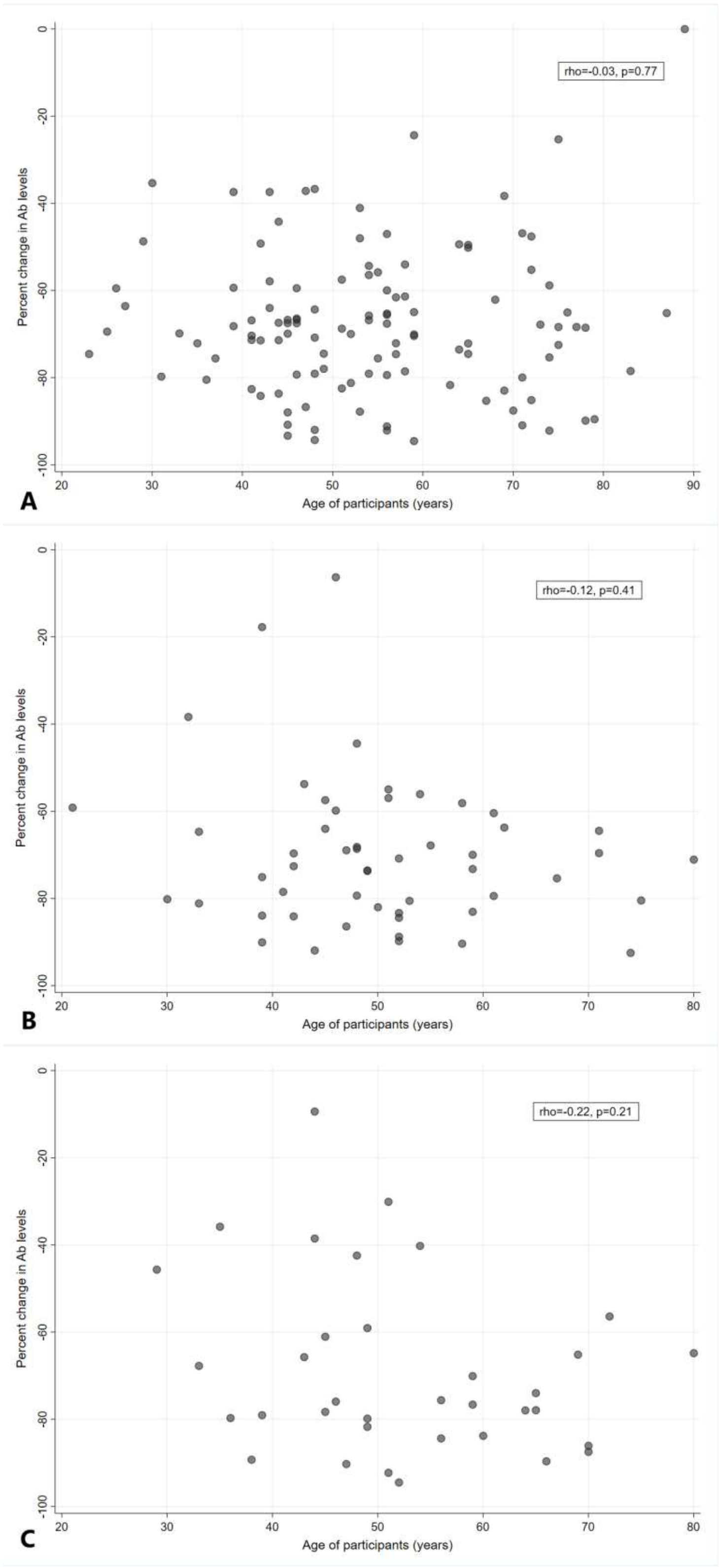

Supplementary Note Fig S5. Correlation between percent change in Ab levels and age of participants, by BNT162b2 (A), BBIBP-CorV (B) and Gam-COVID-Vac (C), after exclusion of the participants with Ab levels above the test's upper limit of quantitation (>400 AU/mL). rho = Spearman's correlation coefficient

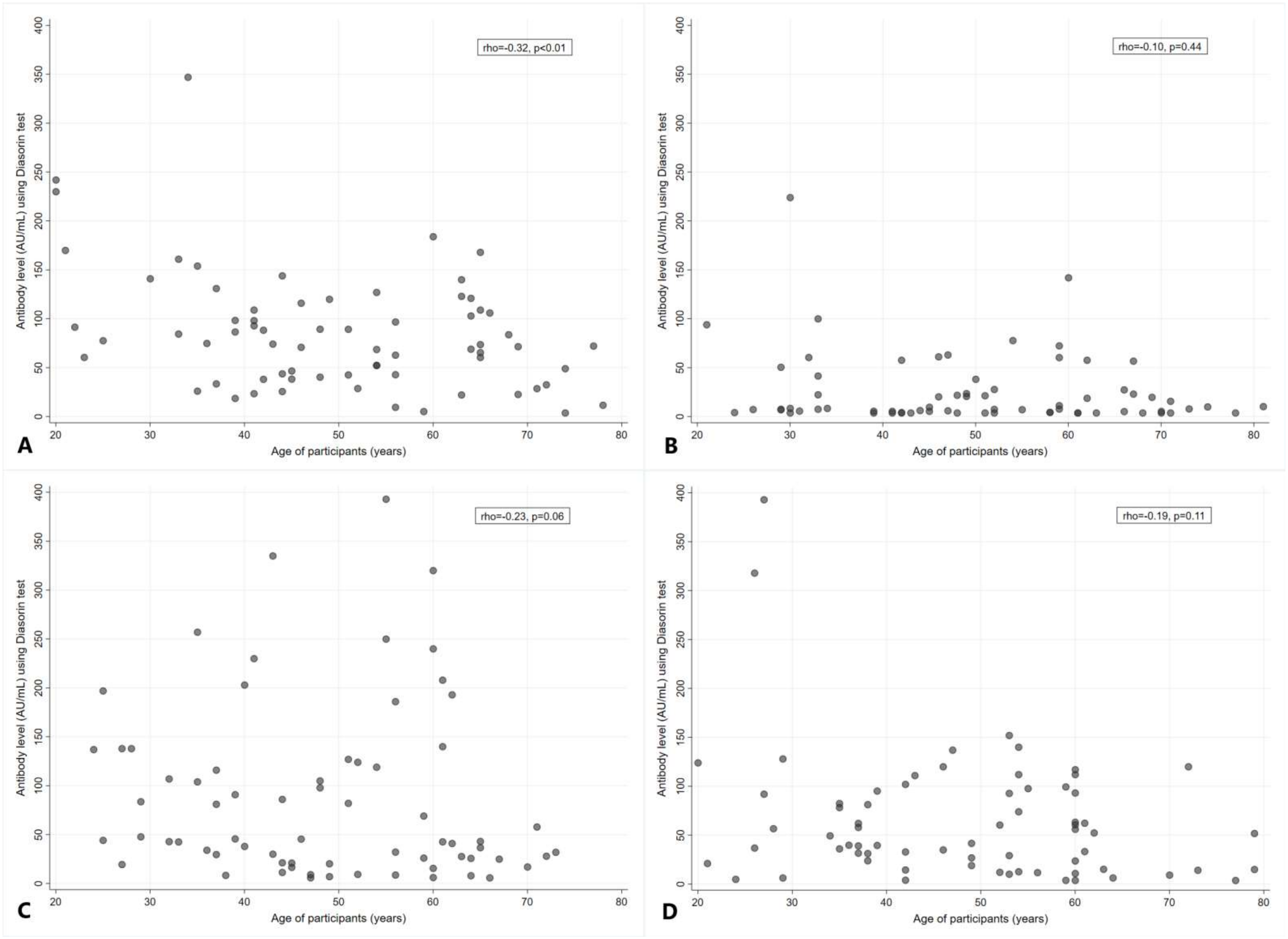

Supplementary Note Fig S6. Correlation between antibody levels measured 6 months post-vaccination after the second dose of BNT162b2 (A), BBIBP-CorV (B), Gam-COVID-Vac (C) and ChAdOx1 nCoV-19(D) vaccines and the age of participants, after exclusion of the participants with Ab levels above the test's upper limit of quantitation (>400 AU/mL). rho = Spearman's correlation coefficient

Supplementary Note Table 1. Antibody levels on 28th day and 6 months after administration of the second dose of vaccine, stratified by sex and age groups of the participants, after exclusion of the participants with Ab levels above the test's upper limit of quantitation (>400 AU/mL).

Vaccine	At 28th day after the second dose							At 6 months after the second dose					
	N	%	Mean (AU/mL)	SD	Median (AU/mL)	IQR (25-75)		Mean (AU/mL)	SD	Median (AU/mL)	IQR (25-75)		p-value[1]
**BNT162b2**													
**Total**	116	100	194.29	88.88	196.5	122.5	248.5	65.37	44.25	60.6	33.05	89.55	**<0.001**
**Sex**													
Female	70	60.34	206.26	92.9	202.5	136	280	72.09	47.64	68.7	36	96.8	**<0.001**
Male	46	39.66	176.08	79.95	176	120	231	55.14	36.71	49.9	32.5	79.2	**<0.001**
**Age category**													
20-29	5	4.31	255.40	93.92	254.00	238.00	259.00	90.90	34.18	77.70	67.20	105.00	0.063
30-39	9	7.76	234.94	91	218	184	297	85.37	52.46	84.5	37.3	98.4	**0.008**
40-49	35	30.17	186.4	92.24	174	118	241	62.96	50.39	46.7	23.5	92.9	**<0.001**
50-59	34	29.31	216.95	77.67	211.5	157.00	281.00	72.57	39.42	67.35	47.70	96.8	**<0.001**
60-69	11	9.48	202.24	101.76	217	121	257	72.65	46.91	71.6	22.7	103	**0.003**
≥70	22	18.97	137.32	65.34	131.00	97.80	199.00	40.47	27.48	35.80	12.70	60.60	**<0.001**
**BBIBP-CorV**													
**Total**	52	100	66.43	66.17	47.8	22.5	78.25	20.6	26.65	7.95	4.3	21.65	**<0.001**
**Sex**													
Female	33	63.46	62.33	66.06	44.1	22.30	72.2	18.91	27.52	7.45	4.37	20.3	**<0.001**
Male	19	36.54	73.55	67.55	51.80	22.70	95.20	23.52	25.52	10.00	3.79	38.20	**<0.001**
**Age category**													
20-29	1	1.92	230.00	..	230.00	..	..	94.00	..	94.00	..	..	NA
30-39	8	15.38	85.88	86.65	48.9	37.45	108.05	36.07	42.91	8.05	5.53	78.8	**0.008**
40-49	18	34.62	50.87	46.57	46.10	13.90	69.70	16.08	18.19	7.98	4.22	20.70	**<0.001**
50-59	16	30.77	73.39	72.96	46.40	23.50	83.95	19.05	24.01	7.61	4.39	24.60	**<0.001**
60-69	4	7.69	43.37	38.17	35.10	13.99	72.75	12.37	10.06	11.30	3.79	20.95	0.125
≥70	5	9.62	54.74	54.59	51.10	13.10	51.60	8.94	4.99	10.00	4.23	11.00	0.063
**Gam-COVID-Vac**													
**Total**	35	100	117.64	69.3	103	60.8	162	40.15	48.14	26.1	9.44	43.1	**<0.001**
**Sex**													
Female	24	68.57	110.87	74.16	92.95	60.1	146.5	36.66	52.23	21.85	13.02	32.1	**<0.001**
Male	11	31.43	132.41	57.71	141	60.8	189	47.79	38.87	42.6	7.12	83.7	**0.003**

**Age category**													
20-29	1	2.86	154.00	..	154.00	..	..	83.70	..	83.70	..	..	NA
30-39	5	14.29	151.64	51.38	162.00	132.00	168.00	46.96	35.09	42.60	34.10	45.70	0.063
40-49	11	31.43	91.94	55.6	87.8	42.6	140	34.02	30.68	21.3	16.6	45.5	**0.003**
50-59	8	22.86	150.01	100.32	114.3	88.25	185	60.38	87.83	24.35	9.1	75.6	**0.008**
60-69	6	17.14	95	63.76	79.7	38.10	141	22.63	17.65	21.98	6.18	36.7	**0.031**
≥70	4	11.43	105.90	39.49	103.20	74.30	137.50	23.48	6.31	23.60	18.10	28.85	0.125

Notes: [1]Wilcoxon matched-pairs signed-rank test (exact test, where applicable), p-value refers to difference between matched groups at two time points of measurements within the same vaccine. In bold are presented statistically significant at $p<0.05$. NA = not applicable, SD = standard deviation, IQR = interquartile range.

Supplementary Note Table 2. Percent change in Ab levels between 28th day and at 6 months, stratified by sex and age groups, after exclusion of the participants with Ab levels above the test's upper limit of quantitation (>400 AU/mL).

	**BNT162b2 vaccine**					**BBIBP-CorV vaccine**					**Gam-COVID-Vac vaccine**				
	**Mean (SD), %-change**	**Median, %-change**	**IQR (25-75)**		**p-value[1]**	**Mean (SD), %-change**	**Median, %-change**	**IQR (25-75)**		**p-value[1]**	**Mean (SD), %-change**	**Median, %-change**	**IQR (25-75)**		**p-value[1]**
**Total**	-67.46 (16.71)	-68.61	-79.17	-59.39	NA	-69.95 (17.01)	-71.82	-81.55	-62.05	NA	-68.74 (20.31)	-75.93	-83.78	-59.05	NA
**Sex**															
**Female**	-66.68 (16.16)	-69.61	-79.07	-57.48	0.50	-71.08 (14.88)	-69.64	-80.51	-63.99	0.98	-68.90 (21.23)	-77.27	-84.08	-60.04	0.78
**Male**	-68.64 (17.63)	-68.34	-81.65	-64.32		-68.00 (20.50)	-73.66	-81.98	-57.42		-68.41 (19.14)	-75.61	-79.83	-45.65	
**Age category**															
**20-29**	-63.13 (9.89)	-63.54	-69.41	-59.46	0.84	-59.13	-59.13	..	..	0.69	-45.65	-45.65	..	..	0.72
**30-39**	-64.20 (17.03)	-69.82	-75.55	-59.34		-66.37 (25.37)	-77.60	-82.51	-51.50		-70.31 (20.74)	-79.04	-79.70	-67.73	
**40-49**	-70.01 (15.52)	-70.33	-82.59	-64.32		-66.72 (19.14)	-69.28	-78.46	-59.80		-62.01 (23.86)	-65.72	-79.83	-42.41	
**50-59**	-67.19 (15.07)	-67.17	-78.53	-57.48		-74.37 (12.67)	-76.86	-83.85	-62.95		-70.46 (23.47)	-76.1	-88.31	-55.13	
**60-69**	-65.4 (16.27)	-72.12	-81.65	-49.52		-69.71 (9.10)	-69.53	-77.37	-62.05		-78.06 (8.37)	-77.92	-83.78	-73.97	
**≥70**	-67.18 (22.48)	-68.43	-85.11	-58.80		-75.60 (11.06)	-71.07	-80.43	-69.57		-73.68 (15.50)	-75.44	-86.76	-60.60	

Note: [1]Wilcoxon rank-sum or Kruskal–Wallis test; p-value refers to difference between the groups of participants vaccinated with the same vaccine, in bold statistically significant p<0.05; SD=standard deviation, IQR = interquartile range, NA = not applicable.

Supplementary Note Table 3. Antibody levels at 6 months from the administration of the second dose of vaccine, stratified by sex and age of the participants, after exclusion of one participant with Ab level above the test's upper limit of quantitation (>400 AU/mL) and three age- and sex-matching participants.

	BNT162b2 vaccine				BBIBP-CorV vaccine				Gam-COVID-Vac vaccine				ChAdOx1 nCoV-19 vaccine				
	**N (%)**	**Mean (SD), AU/mL**	**Median (IQR, 25-75), AU/mL**	**p-value[1]**	**N (%)**	**Mean (SD), AU/mL**	**Median (IQR, 25-75), AU/mL**	**p-value[1]**	**N (%)**	**Mean (SD), AU/mL**	**Median (IQR, 25-75), AU/mL**	**p-value[1]**	**N (%)**	**Mean (SD), AU/mL**	**Median (IQR, 25-75), AU/mL**	**p-value[1]**	**p-value[2]**
**Total**	69 (100)	85.33 (60.45)	74.20 (42.70-109.00)	NA	69 (100)	25.48 (37.14)	7.91 (4.37-27.40)	NA	69 (100)	86.79 (88.95)	44.20 (25.00-124.00)	NA	69 (100)	63.85 (65.70)	49.40 (19.00-93.20)	NA	**<0.01**
**Sex**																	
Female	35 (50.72)	81.47 (47.87)	77.70 (43.80-103.00)	0.90	35 (50.72)	24.86 (42.68)	7.59 (4.22-22.40)	0.54	35 (50.72)	90.44 (103.47)	42.70 (20.30-137.00)	0.59	35 (50.72)	68.12 (69.11)	52.40 (29.20-97.70)	0.41	**<0.01**
Male	34 (49.28)	89.31 (71.68)	69.95 (38.20-123.00)		34 (49.28)	26.11 (31.06)	9.22 (5.20-41.60)		34 (49.28)	83.02 (72.42)	57.85 (29.70-119.00)		34 (49.28)	59.45 (62.72)	40.65 (14.50-93.20)		**<0.01**
**Age category**																	
20-29	6 (8.70)	145.30 (79.72)	130.75 (77.70-230.00)	**<0.01**	6 (8.70)	28.44 (36.64)	7.45 (7.04-50.50)	0.68	8 (11.59)	100.65 (61.23)	110.35 (46.00-138.00)	0.33	10 (14.49)	118.10 (133.92)	74.35 (21.10-128.00)	0.62	0.06
30-39	12 (17.39)	113.06 (88.31)	92.50 (54.20-147.50)		12 (17.39)	40.99 (64.81)	8.47 (5.65-51.05)		12 (17.39)	79.95 (65.90)	63.40 (38.35-105.50)		14 (20.29)	53.44 (22.88)	44.60 (36.10-78.30)		**0.01**
40-49	17 (24.64)	74.10 (36.76)	74.20 (40.40-98.20)		17 (24.64)	18.87 (21.19)	6.30 (4.22-21.90)		17 (24.64)	75.49 (94.79)	30.10 (16.60-97.90)		11 (15.94)	58.55 (48.52)	35.00 (19.00-111.00)		**<0.01**
50-59	12 (17.39)	56.56 (35.50)	52.40 (35.75-79.00)		14 (20.29)	24.86 (26.81)	9.54 (4.40-38.20)		12 (17.39)	118.89 (113.21)	100.55 (29.15-156.50)		14 (20.29)	64.87 (51.82)	67.20 (12.10-99.40)		**<0.01**

60-69	16 (23.19)	95.19 (45.83)	93.40 (67.25-122.00)		12 (17.39)	30.50 (40.08)	19.30 (3.79-42.10)		16 (23.19)	86.17 (100.15)	38.85 (20.30-166.50)		14 (20.29)	50.73 (37.65)	54.25 (15.20-63.30)		**<0.01**
≥70	6 (8.70)	33.02 (24.99)	30.65 (11.70-49.10)		8 (11.59)	7.58 (4.26)	6.64 (3.79-10.15)		4 (5.80)	33.73 (17.32)	30.00 (22.50-44.95)		6 (8.70)	35.67 (44.66)	14.60 (9.25-51.80)		**0.04**

Note: For statistical processing and presentation of data, results below the minimum detectable value of the assay (<3.8) were interpreted as 3.79. NA=not applicable.[1]Wilcoxon rank-sum or Kruskal–Wallis test; p-value refers to difference between groups within the same vaccine. [2]Kruskal-Wallis test; p-value refers to difference between groups across different vaccines. SD=standard deviation, IQR = interquartile range, NA = not applicable.

Supplementary Note Table 4. Serological profile of the study participants at 6 months from the administration of the second dose of four different SARS-CoV-2 vaccines, after exclusion of one participant with Ab level above the test's upper limit of quantitation (>400 AU/mL) and three age- and sex- matching participants.

	Total sample	BNT162b2 vaccine (n=69)				BBIBP-CorV vaccine (n=69)				Gam-COVID-Vac vaccine (n=69)				ChAdOx1 nCoV-19 vaccine (n=69)			
		**Sero-negative**	**Equi-vocal**	**Sero-positive**	**p-value[1]**	**Sero-negative**	**Equi-vocal**	**Sero-positive**	**p-value[1]**	**Sero-negative**	**Equi-vocal**	**Sero-positive**	**p-value[1]**	**Sero-negative**	**Equi-vocal**	**Sero-positive**	**p-value[1]**
**Total, n (%)**	276 (100)	4 (5.80)	0	65 (94.20)	NA	41 (59.42)	0	28 (40.58)	NA	10 (14.49)	0	59 (85.51)	NA	11 (15.94)	4 (5.80)	54 (78.26)	NA
**Sex, n (%)**																	
Female	140 (100)	2 (5.71)	0	33 (94.29)	1.00	22 (62.86)	0	13 (37.14)	0.56	5 (14.29)	0	30 (85.71)	1.00	4 (11.43)	2 (5.71)	29 (82.86)	0.56
Male	136 (100)	2 (5.88)	0	32 (94.12)		19 (55.88)	0	15 (44.12)		5 (14.71)	0	29 (85.29)		7 (20.59)	2 (5.88)	25 (73.53)	
**Age (years), mean (SD)**	49.93 (15.13)	66.75 (10.87)	NA	48.89 (14.80)	**0.02**	50.32 (15.79)	NA	49 .54 (13.86)	0.83	52.30 (9.06)	NA	47.92 (13.88)	0.34	54.00 (16.30)	55.25 (12.95)	46.59 (14.11)	0.82
**Age category, n (%)**																	
20-29	30 (100)	0	0	6 (100)		4 (66.67)	0	2 (33.33)		0	0	8 (100)		2 (20.00)	0	8 (80.00)	
30-39	50 (100)	0	0	12 (100)		7 (58.33)	0	5 (41.67)		1 (8.33)	0	11 (91.67)		0	0	14 (100)	
40-49	62 (100)	0	0	17 (100)		10 (58.82)	0	7 (41.18)		4 (23.53)	0	13 (76.47)		1 (9.09)	1 (9.09)	9 (81.82)	
50-59	52 (100)	2 (16.67)	0	10 (83.33)	**<0.01**	8 (57.14)	0	6 (42.86)	0.52	2 (16.67)	0	10 (83.33)	0.72	3 (21.43)	2 (14.28)	9 (64.29)	0.17
60-69	58 (100)	0	0	16 (100)		5 (41.67)	0	7 (58.33)		3 (18.75)	0	13 (81.25)		3 (21.43)	0	11 (78.57)	
≥70	24 (100)	2 (33.33)	0	4 (66.67)		7 (87.50)	0	1 (12.50)		0	0	4 (100)		2 (33.33)	1 (16.67)	3 (50.00)	

Note: participants were classified based on the Ab levels at 6 months after vaccination as: seronegative (<12.0 AU/mL), equivocal (12.0 – 15.0 AU/mL), seropositive (>15.0 AU/mL). [1]Pearson's chi-squared test (Fisher's exact test) for categorical and t-test or ANOVA-analysis of variance for continuous variables. Significance levels are given in bold for $p<0.05$. NA = not applicable.
