# Supplementary figures and images for: Immunogenicity of BNT162b2, BBIBP-CorV, Gam-COVID-Vac and ChAdOx1 nCoV-19 Vaccines Six Months after the Second Dose: A Longitudinal Prospective Study
Source: Vaccines (Basel). 2022 Dec 26;11(1):56. doi: 10.3390/vaccines11010056 (PMC9865554; doi:10.3390/vaccines11010056)

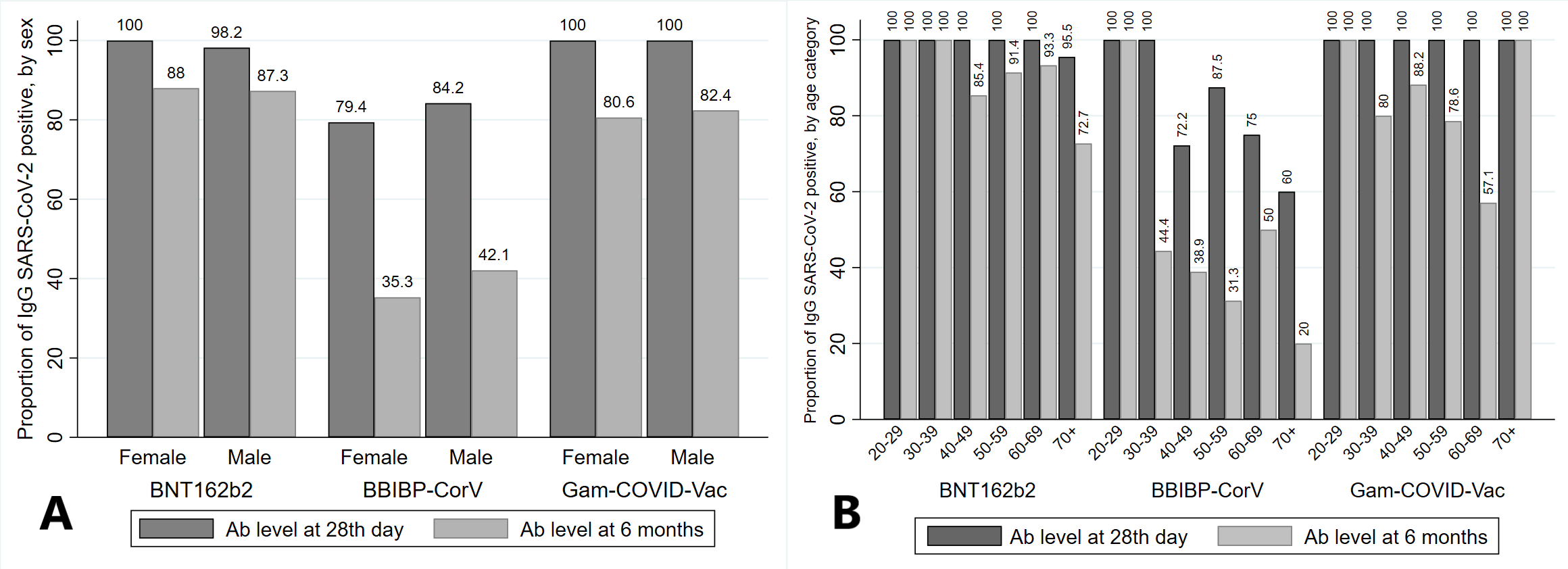

Supplement: Supplementary file 1 [file vaccines-11-00056-s001.zip › FigureS1.tiff]

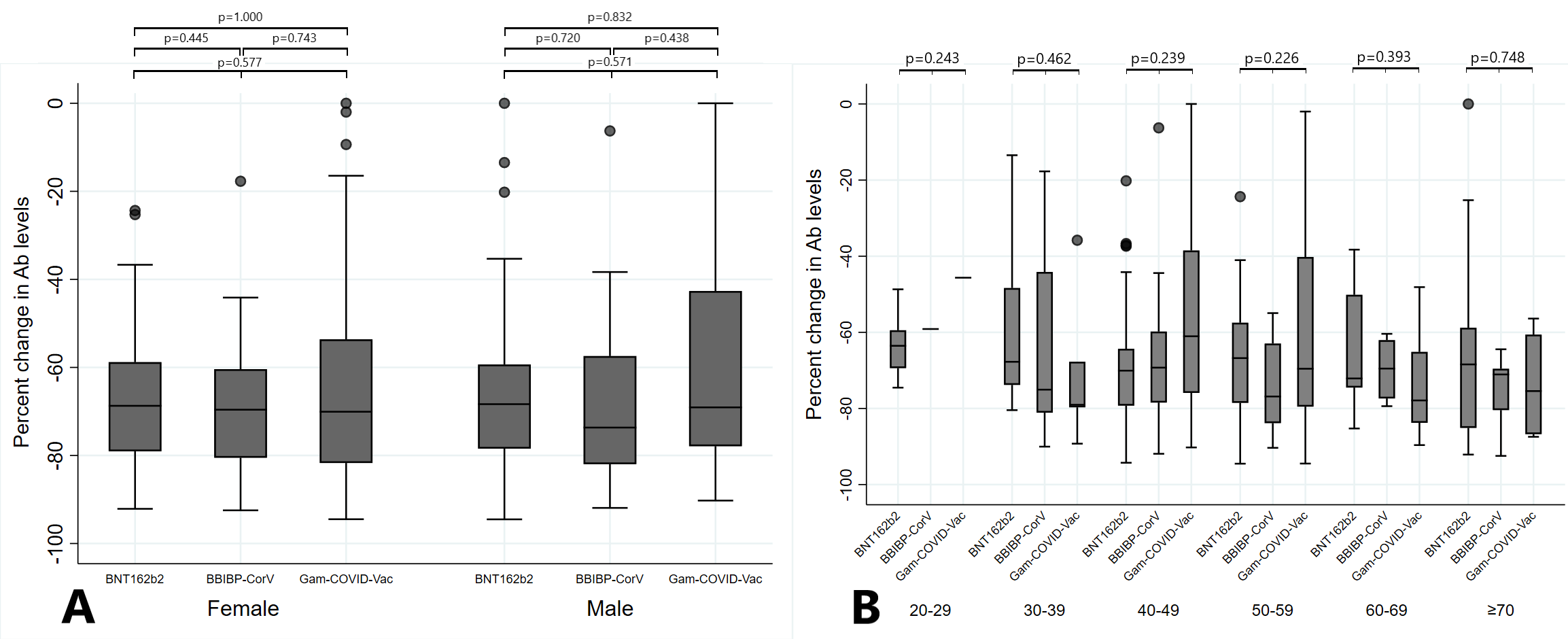

Supplement: Supplementary file 1 [file vaccines-11-00056-s001.zip › FigureS2.tiff]

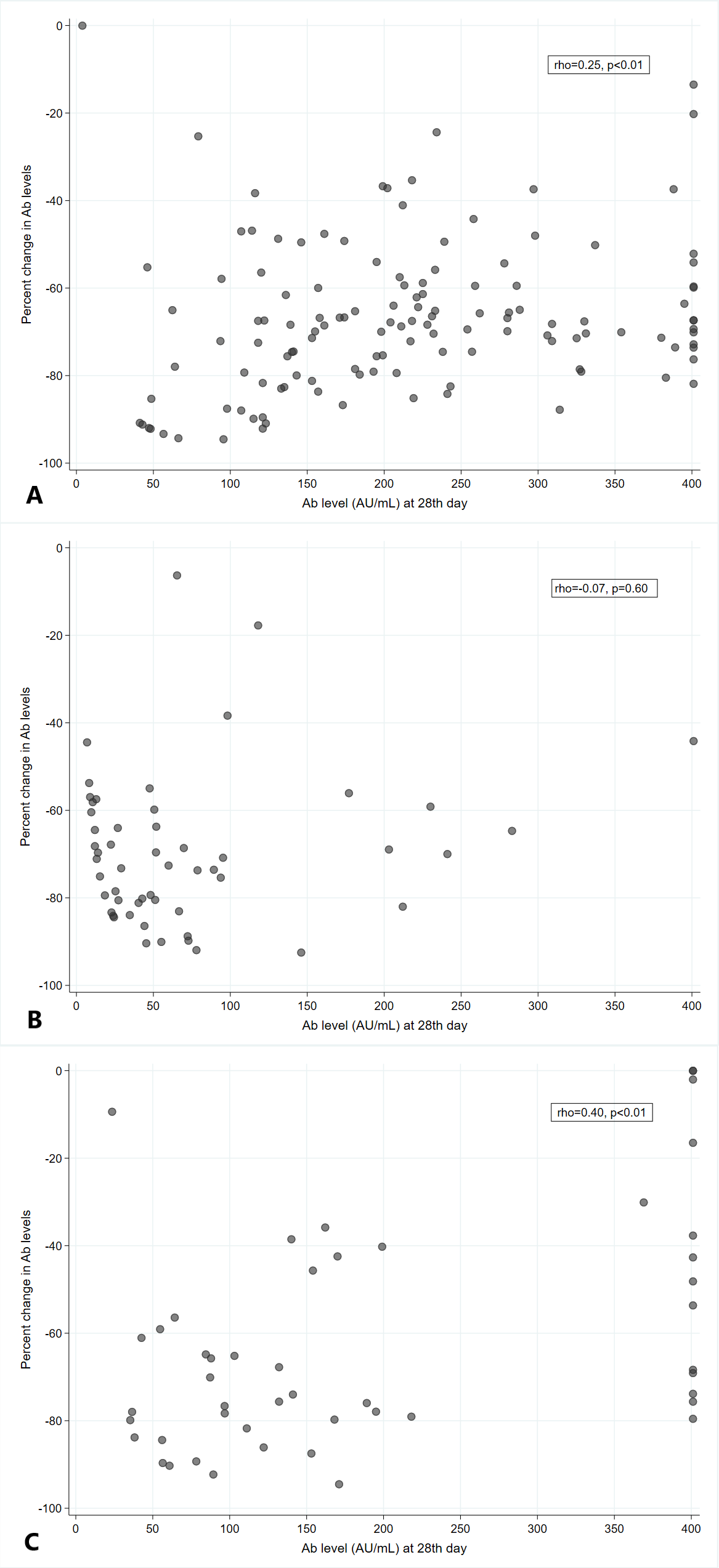

Supplement: Supplementary file 1 [file vaccines-11-00056-s001.zip › FigureS3.tiff]

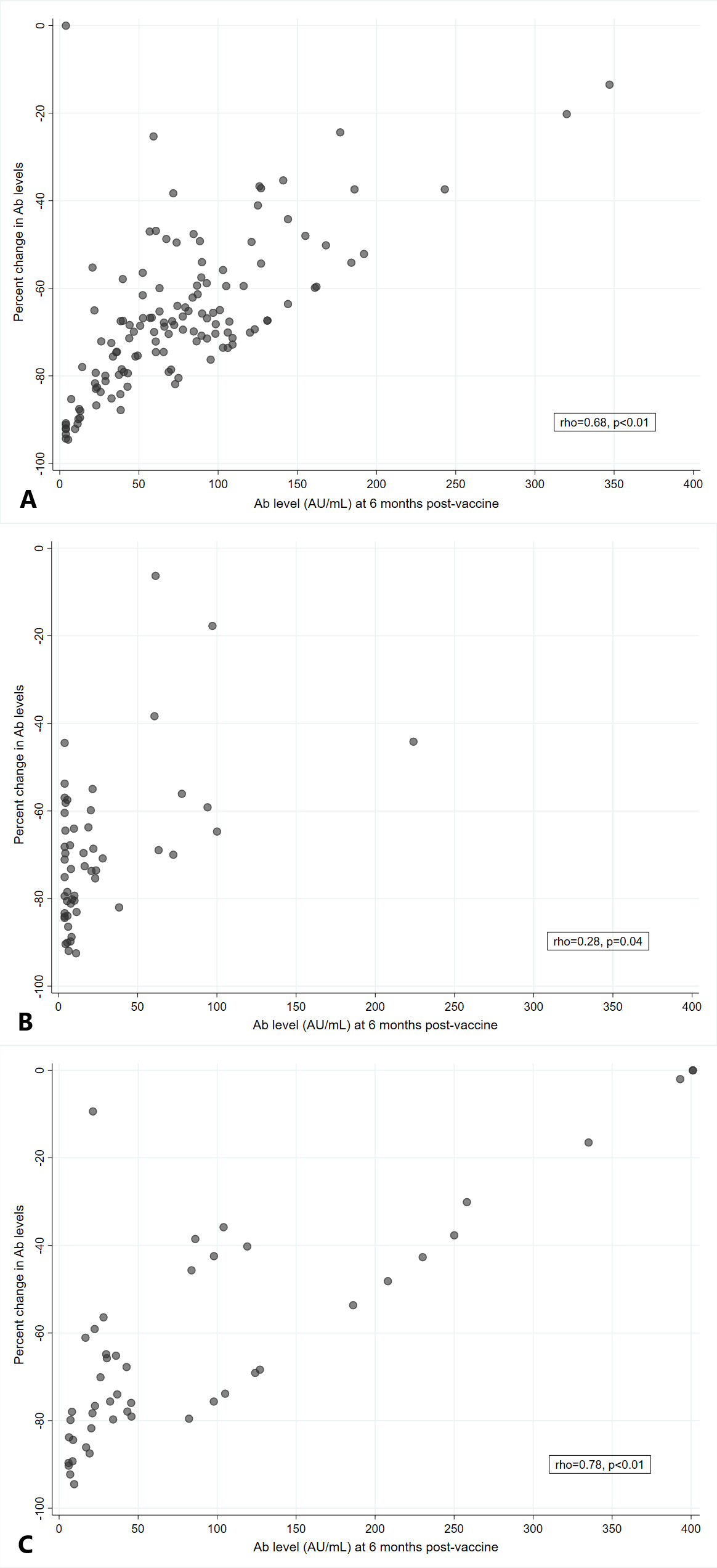

Supplement: Supplementary file 1 [file vaccines-11-00056-s001.zip › FigureS4.tiff]

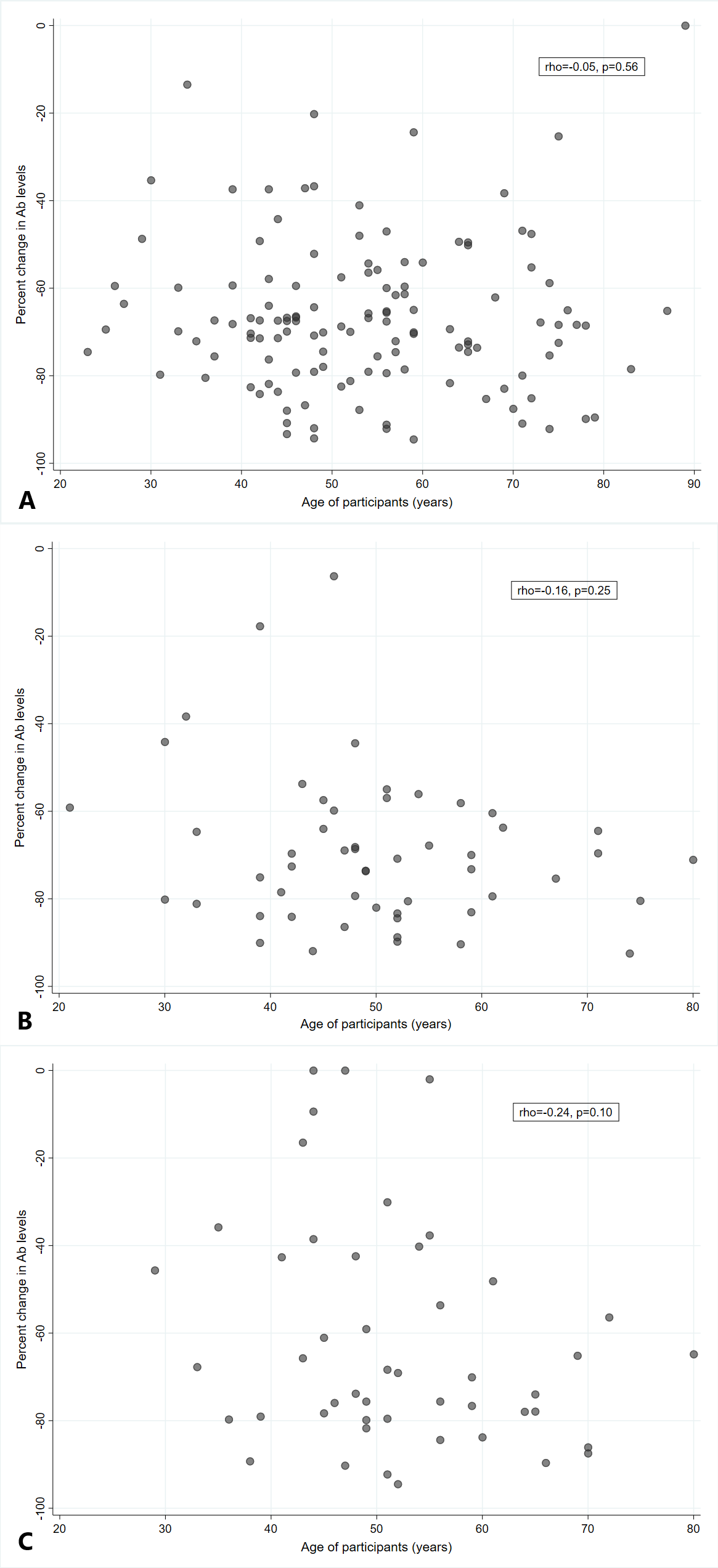

Supplement: Supplementary file 1 [file vaccines-11-00056-s001.zip › FigureS5.tiff]

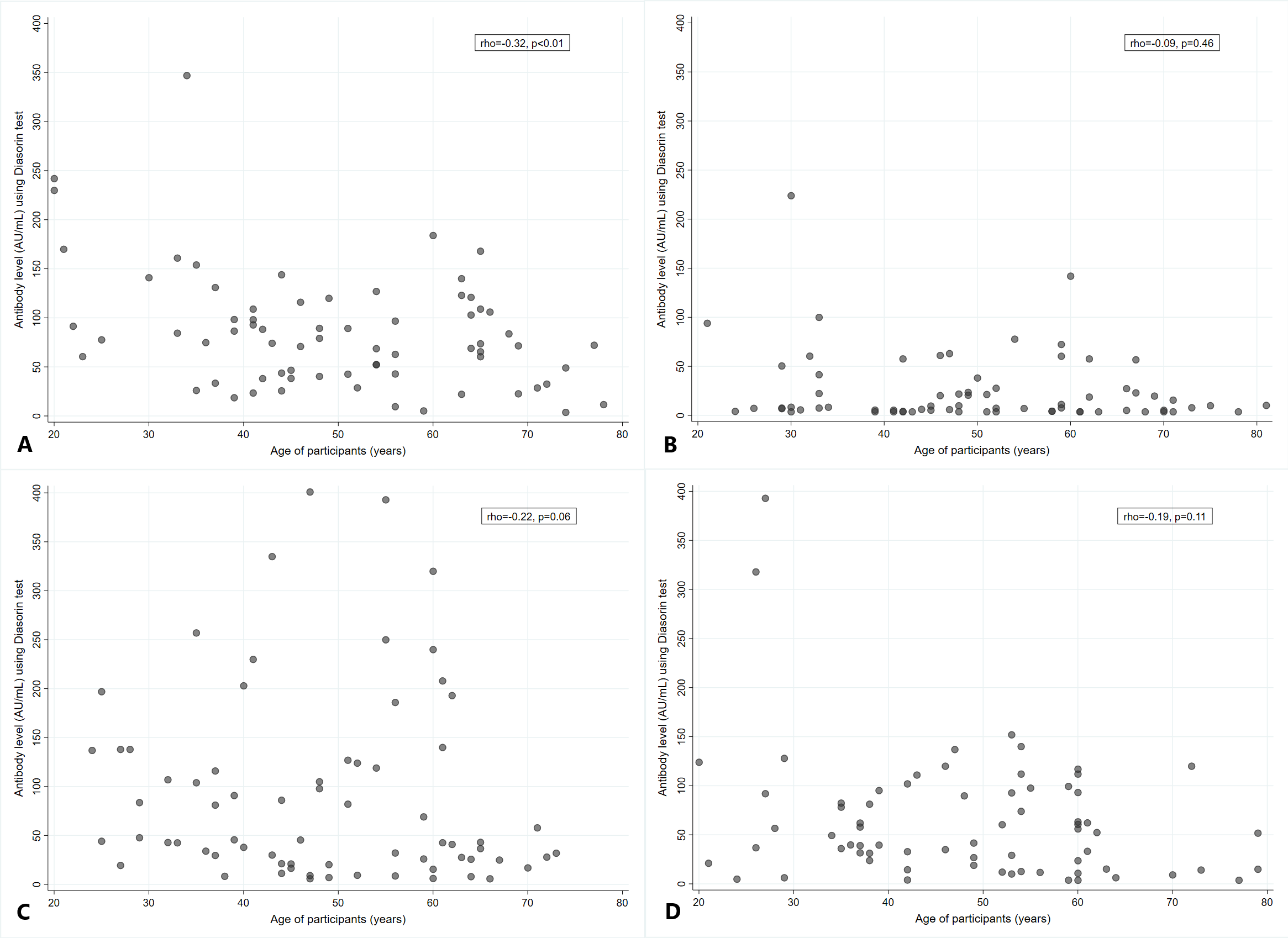

Supplement: Supplementary file 1 [file vaccines-11-00056-s001.zip › FigureS6.tiff]
